# System level modeling and analysis of TNF-*α* mediated sphingolipid signaling pathway in neurological disorders for the prediction of therapeutic targets

**DOI:** 10.3389/fphys.2022.872421

**Published:** 2022-08-19

**Authors:** Sanam Banaras, Rehan Zafar Paracha, Maryum Nisar, Ayesha Arif, Jamil Ahmad, Muhammad Tariq Saeed, Zartasha Mustansar, Malik Nawaz Shuja, Rizwan Nasir Paracha

**Affiliations:** ^1^ School of Interdisciplinary Engineering and Sciences (SINES), National University of Sciences and Technology (NUST), Islamabad, Pakistan; ^2^ Computer Science and Information Technology (CS&IT), University of Malakand, Chakdara, Pakistan; ^3^ Kohat University of Science and Technology, Kohat, Pakistan; ^4^ Department of Chemistry, University of Sargodha, Sub Campus Bhakkar, Bhakkar, Pakistan

**Keywords:** neurological disorders, neurons, microglia, TNF-α, sphingomyelin, network analysis, quantitative analysis, drug repurposing

## Abstract

Sphingomyelin (SM) belongs to a class of lipids termed sphingolipids. The disruption in the sphingomyelin signaling pathway is associated with various neurodegenerative disorders. TNF-*α*, a potent pro-inflammatory cytokine generated in response to various neurological disorders like Alzheimer’s disease (AD), Parkinson’s disease (PD), and Multiple Sclerosis (MS), is an eminent regulator of the sphingomyelin metabolic pathway. The immune-triggered regulation of the sphingomyelin metabolic pathway *via* TNF-*α* constitutes the sphingomyelin signaling pathway. In this pathway, sphingomyelin and its downstream sphingolipids activate various signaling cascades like PI3K/AKT and MAPK/ERK pathways, thus, controlling diverse processes coupled with neuronal viability, survival, and death. The holistic analysis of the immune-triggered sphingomyelin signaling pathway is imperative to make necessary predictions about its pivotal components and for the formulation of disease-related therapeutics. The current work offers a comprehensive in silico systems analysis of TNF-*α* mediated sphingomyelin and downstream signaling cascades *via* a model-based quantitative approach. We incorporated the intensity values of genes from the microarray data of control individuals from the AD study in the input entities of the pathway model. Computational modeling and simulation of the inflammatory pathway enabled the comprehensive study of the system dynamics. Network and sensitivity analysis of the model unveiled essential interaction parameters and entities during neuroinflammation. Scanning of the key entities and parameters allowed us to determine their ultimate impact on neuronal apoptosis and survival. Moreover, the efficacy and potency of the FDA-approved drugs, namely Etanercept, Nivocasan, and Scyphostatin allowed us to study the model’s response towards inhibition of the respective proteins/enzymes. The network analysis revealed the pivotal model entities with high betweenness and closeness centrality values including recruit FADD, TNFR_TRADD, act CASP2, actCASP8, actCASP3 and 9, cytochrome C, and RIP_RAIDD which profoundly impacted the neuronal apoptosis. Whereas some of the entities with high betweenness and closeness centrality values like Gi-coupled receptor, actS1PR, Sphingosine, S1P, actAKT, and actERK produced a high influence on neuronal survival. However, the current study inferred the dual role of ceramide, both on neuronal survival and apoptosis. Moreover, the drug Nivocasan effectively reduces neuronal apoptosis *via* its inhibitory mechanism on the caspases.

## 1 Introduction

Neurological disorders refer to the diseases of the central and peripheral nervous systems that affect the brain, spinal cord, or nerves connecting them. Some of the major neurological disorders include Alzheimer’s disease (AD), Parkinson’s disease (PD), Multiple sclerosis (MS), and various traumatic central nervous system (CNS) injuries (Chen et al., 2016). Patients’ conditions worsen when neurological illnesses are neurodegenerative, resulting in excessive neuronal death and loss. Alzheimer’s disease is an incurable, chronic, multifactorial neurodegenerative disorder that ultimately leads to dementia. (Kumar and Singh, 2015). It is the sixth leading cause of death in the US with 121,499 deaths during the year 2019 (Alzheimer’s Association 2021). Memory loss, language difficulty, depression, lack of intellectual coordination, and agitation are all common psychological and behavioral symptoms of AD. (Haque et al., 2019). It occurs due to the abnormal accumulation of amyloid *β*-protein in the multiple regions of the brain. Correspondingly, Parkinson’s disease is degenerative neuropathological state characterized by prominent neuronal death in substantia nigra (Siciliano et al., 2018). Substantia nigra is midbrain region responsible for modulating movement and maintaining balance (Eriksen et al., 2009). The general symptoms of PD include resting tremor, stiffness of limbs and trunk, bradykinesia, sleeping disorder, speech difficulties, and postural instability (Lee and Yankee, 2021). Current statistics show the second highest prevalence of PD among the neurodegenerative diseases (Orozco et al., 2020). Multiple Sclerosis is autoimmune neurodegenerative disorder causing demyelination of CNS. The disease targets young adults and has an increasing prevalence worldwide (McGinley et al., 2021).

The microglial cells are the resident immune cells in the brain parenchyma, dispersed throughout the CNS in a non-heterogeneous manner and constitute 10% of the total glial population. Glial cells are non-neuronal cells within the central (CNS) and peripheral nervous system (PNS) responsible for neuronal protection, development, immunity, and homeostasis (Li et al., 2020; Basham, 2021). In neurodegenerative disorders like AD, PD, and MS, the loss of neurons owing to pathogenic insults causes activation and aggregation of microglia around affected brain regions. (Ho, 2019). The activated microglia generate a pro-inflammatory response by releasing several pro-inflammatory mediators known as cytokines, including tumor necrosis factor-*α* (TNF-*α*), Interleukin (IL)-1*β*, and IL-16, which aggravate neuronal degeneration (Kwon and Koh, 2020). These cytokines further amplify the inflammatory response by recruiting other immune cells to the affected site (Carniglia et al., 2017). Neuronal activities such as calcium homeostasis, membrane potential, sleep, synapses, learning, and memory are all regulated by TNF-*α* in the healthy CNS. Moreover, as discussed earlier, TNF-*α* is a proinflammatory mediator involved in generating an innate immune response associated with various neurological disorders. To wit, under normal circumstances, the levels of TNF-*α* consistently regulate the physiological processes under its influence. TNF-*α*, which is released by astrocytes and microglia during pathological conditions such as AD, PD, and MS is a prominent component of the neuroinflammatory response (Olmos and Lladó, 2014).

Sphingolipids are a class of lipids highly enriched in the central nervous system (CNS), and they are essential for the development and maintenance of its functional integrity. TNF-*α* can supervise neuronal survival and apoptosis by modulating the sphingolipid signal transduction pathway at various levels. The process starts with the potential binding of TNF-*α* binds with two of its receptors, namely, tumor necrosis factor receptor 1 (TNFR1) and tumor necrosis factor receptor 2 (TNFR2) (Cabal-Hierro and Lazo, 2012). TNFR1 constitutively and ubiquitously expresses across all human tissues, including the brain, whereas TNFR2 expresses primarily in neurons and immune cells. However, both the receptors differ in their intracellular death domain (DD) regions. TNFR1 possesses the death domain (DD) on its cytoplasmic tail, whereas TNFR2 lacks this motif. The binding of TNF-*α* with TNFR1 triggers the initiation of the TNF-*α* signaling pathway. The downstream modulators of TNFR1 activation like TRADD and FADD directly or indirectly mediate the number of signaling cascades besides producing apoptotic effects (Wajant and Siegmund, 2019). The modulation of nuclear factor-kappa B (NF-*κ* B) and sphingomyelin signal transduction pathways *via* TNF-*α* implicates the role of TNF-*α* in neuronal death and survival (Malagarie-Cazenave et al., 2002). Besides, the sphingomyelin (SM) signaling pathway establishes a distinct linkage between TNF-*α*, PI3K/AKT, and MAPK/ERK pathways. In neuropathological conditions like AD, PD, and MS, microglia and astrocytes release large amounts of TNF-*α* that alter its usual physiological and regulatory effects (Subhramanyam et al., 2019). Therefore, it can be assumed that the attainment of neuronal survival and apoptotic equilibrium depends on the TNF-*α* concentration in the brain.

Several disease-specific immunization strategies and vaccines has been in clinical studies to boost immune responses against proteins that cause pathological changes in neuroinflammatory disorders. For instance, immunization of transgenic mouse models with *β*-amyloid has given some productive results in the attenuation of Alzheimer-like pathology (Schenk et al., 1999). In the case of AD, Aducanumab is approved as the first FDA-approved therapy which halts the adverse effects of Alzheimer’s by reducing *β*-amyloid from the brain to treat AD patients (Esang and Gupta, 2021). Similarly, PD medications simply alleviate symptoms but do not prevent the condition from deteriorating. The holistic system analysis of the model of immune-triggered pathways presents a realistic approach for making critical predictions regarding pathway components and disease-related treatments. However, the system analysis of static biological pathways is ineffective compared to the system-level dynamic analysis of pathways. Qualitative and Quantitative Analysis are the two standard methodologies used for dynamic system analysis of biological pathways.

Previously, Cho et al., (2003) studied a mathematical model of the TNF-*α*-mediated NF-*κ*B pathway (Cho et al., 2003). Using the Monte Carlo method, sensitivity analysis of model parameters was performed to identify key proteins and parameters. In two quite a similar studies, the mathematical model of TNF-*α* initiated survival and apoptotic cascades were designed using mass-action kinetics. The model dynamics and results were compared with the experimental findings (Rangamani and Lawrence, 2007; Chignola et al., 2009). Moreover,Alvarez-Vasquez et al., (2004) constructed integrative dynamical mathematical models for sphingolipid metabolism in yeast using kinetic information from literature for the first time (Alvarez-Vasquez et al., 2004). In another study conducted for *Saccharomyces cerevisiae*, a mathematical model of sphingolipid metabolism using flux-balance analysis was designed and validated experimentally. Simulations of the model precisely manifested the effect of various perturbations that were in exact accordance with the post hoc experimental findings (Alvarez-Vasquez et al., 2004). Furthermore, in another study, the sphingolipid metabolic pathway was analyzed to study the control aspects of the diauxic shift in yeast (Alvarez-Vasquez et al., 2007). Likewise, various computational modeling techniques have been employed to model pathological signaling in several neurodegenerative disorders. A comparatively recent and advanced study proposed a comprehensive computational model for the sphingolipid metabolism (Wronowska et al., 2015). The study used several model analysis methods to detect sensitive and experimentally non-identifiable parameters in the sphingolipid pathway. Also, the model was employed to understand the underlying molecular mechanisms of the sphingolipid metabolism in AD. A recent computational study explored the role of network dynamics towards the initiation and progression of AD (Cutsuridis and Moustafa, 2017).

The current study employs a model-based quantitative approach to conduct in silico systems analysis of TNF-*α* induced sphingomyelin and downstream signaling cascades. To perform quantitative systems analysis, we build a model that depicts TNF-*α* effect on the sphingolipid signaling pathway and incorporates gene intensity values from microarray analysis. The inflammatory process may be studied thoroughly using computational modeling and simulation. The model’s network and sensitivity analyses reveal the importance of key interaction parameters and entities in the advanced phases of neuroinflammation. Furthermore, we assess the efficacy and potency of FDA-approved medicines, namely Etanercept, Nivocasan, and Scyphostatin, at later phases of neuronal inflammation by analyzing the model’s response to protein/enzyme inhibition. The study identifies new biomarkers, essential and novel system components such as TNFR adaptor proteins or sphingolipids as effective and potential drug targets at inflammatory stages, and investigates drug repurposing.

## 2 Methodology

**FIGURE 1:**
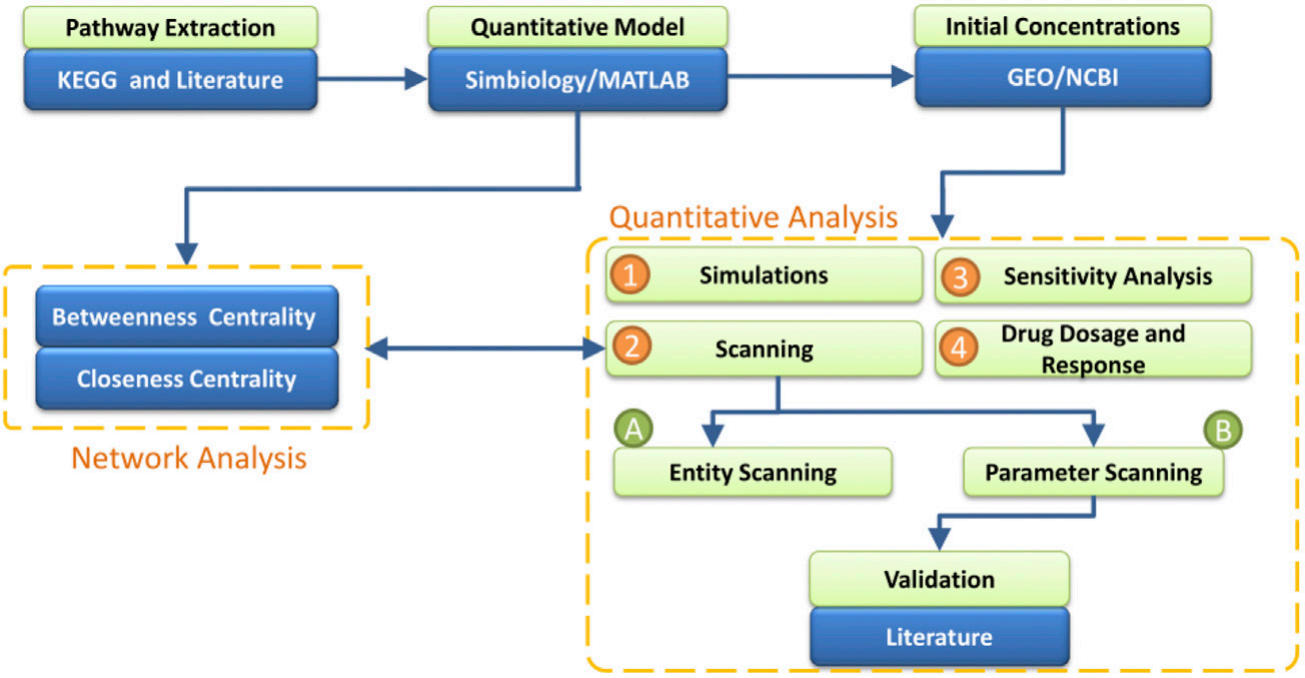
Work-Flow of the study- The analysis starts with the extraction of TNF-*α* mediated sphingomyelin signaling pathway information from literature and KEGG. The step is followed by the construction of the model of extracted pathways in Simbiology. The model is subjected to two forms of analysis: Network Analysis and Quantitative Analysis. The network analysis includes two measures: Betweenness Centrality and Closeness Centrality which help to identify the essential proteins in the model. For the quantitative analysis, the initial concentrations of entities are incorporated in the model from the microarray gene expression data of normal individuals. The measures of quantitative analysis include Simulation of the model’s entities, Scanning of entities and parameters, Sensitivity Analysis of entities vs. entities and parameters vs. entities, and the effect of different Drugs’ Dosages (Etanercept, Nivocasan, and Scyphostatin) over the model. Moreover, the results from Network Analysis and Quantitative Analysis are compared.

The current study offers a computational workflow based on the quantitative modeling of the TNF-*α* mediated sphingomyelin signaling pathway and its downstream signaling pathways (MAPK and PI3K-AKT). The model was generated in Matlab/Simbiology, based on the data from the literature as well as the KEGG database. The gene expression data from a microarray dataset of AD study were taken as the initial values of the input entities of the model. The transitions of the model were adjusted with the kinetic rates based on the law of mass action. Network analysis and quantitative analyses were then performed, which allowed us to identify and analyze the key entities and interactions of the model. Eventually, the drug-dependent signaling in the model enabled studying the effects of different dosages of drugs and corresponding model responses (Figure 1).

### 2.1 Pathway extraction

**FIGURE 2:**
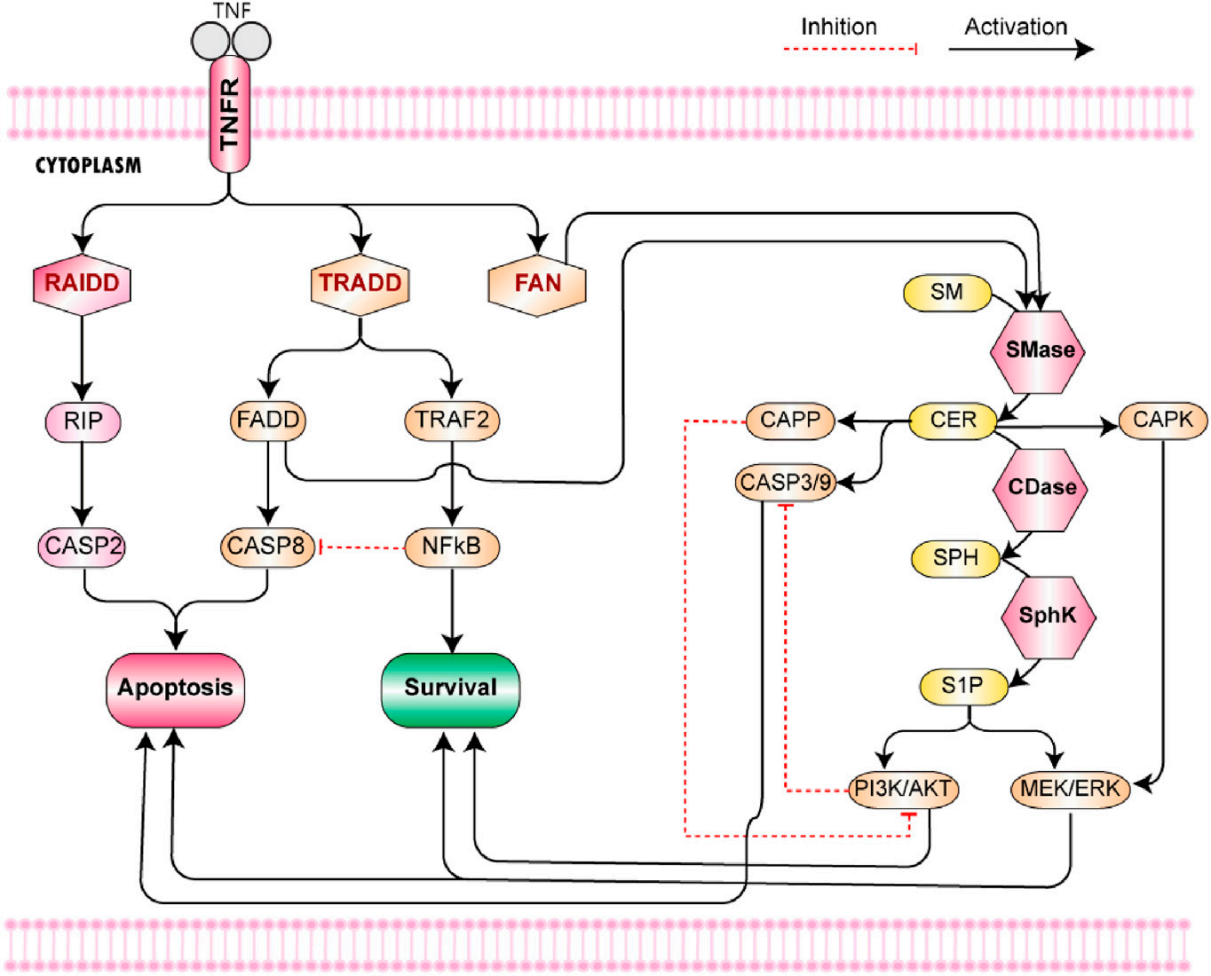
TNF-*α* mediated Sphingomyelin Signaling Pathway- The pathway starts from the binding of TNF-*α* with TNF receptor 1. The binding of TNF-*α* trimer with TNFR1 leads to receptor trimerization. It is followed by the recruitment of three cytosolic adaptor proteins, namely, TRADD, RAIDD, and FAN. TRADD and RAIDD bind with the DD of TNFR1 while FAN binds with its NSD. The recruitment of TRADD causes the recruitment of three additional adapter proteins, namely, RIP, TRAF2, and FADD. The RIP along with RAIDD (another death domain-containing molecule) induces activation of caspase 2, which leads to apoptosis. FADD allows the activation of caspase 8 *via* interacting with its DED domain (death effector domain). TRAF2 is capable of interacting with NIK (NF-*κ*B Inducing Kinase) which belongs to the MEKK family. NIK phosphorylates its target IKK (Inhibitor of *κ*B Kinase) which ultimately leads to the activation of NF-*κ*B. NF-*κ*B produces an anti-apoptotic effect by promoting transcription of cellular inhibitor of apoptosis protein 2 (c-IAP2), which blocks the caspase 8 activation and consequently apoptosis. In the sphingolipid metabolic pathway, sphingomyelinases (SMase) catalyze the conversion of sphingomyelin to ceramide. In neurons, ceramide is produced by the hydrolysis of sphingomyelin *via* acidic (A-SMase) or neutral (N-SMase). The activation of both forms of SMases is mediated by the TNF-*α* signaling cascade. The adaptor proteins TRADD and FADD induce the activity of acid SMase, whereas FAN is associated with the activation of neutral SMase. Ceramide, generated in response to acid sphingomyelinase, activates aspartyl protease cathepsin D responsible for the cleavage and activation of pro-apoptotic Bid (family member Bcl-2). The elevated levels of ceramide cause increased permeability of mitochondria which induces the release of cytochrome C from the mitochondria. It causes the activation of caspases 3 and 9, ultimately leading to apoptosis *via* an intrinsic pathway. On the other hand, ceramide produced *via* neutral sphingomyelinase (nSMase) directly activates two target enzymes, namely, CAPK (ceramide activated protein kinase) and CAPP (ceramide activated protein phosphatase) (Haughey et al., 2010). Both of them are capable of evoking signaling that leads to apoptosis. CAPK phosphorylates raf-1, which enhances its activity towards MEK (MAPK kinase). The MAPK signaling cascade induces activation of ERK cascade, which either results in cell cycle arrest and cell death or contributes to neuronal cell survival depending upon the form of ERK activated. On the other hand, CAPP (comprising of serine-threonine phosphatases PP1 and PP2A) moves the system towards apoptosis by inactivating Akt.

KEGG is a pathway database, which provides the metabolic, genomic, and chemical information for understanding high-level functions of the biological systems (Kanehisa and Goto, 2000). To model TNF-*α* mediated sphingomyelin and downstream signaling pathways, we extracted the pathway data from the KEGG pathway database as well as literature (Supplementary Figure S1). TNF-*α* initiates the pathway *via* binding with TNF receptors (TNFR1 and TNFR2) (Figure 2). TNFR1 possesses the death domain (DD) on its cytoplasmic tail, whereas TNFR2 lacks this motif Ruiz et al., (2021). The binding of TNF-*α* trimer with TNFR1 leads to the receptor trimerization followed by the recruitment of cytosolic adaptor protein called TRADD. The recruitment of TRADD causes the recruitment of three additional adapter proteins, namely, receptor-interacting protein (RIP), TNF receptor-associated factor 2 (TRAF2), and Fas-associated death domain (FADD). The RIP along with RAIDD (another death domain-containing molecule) induces activation of caspase 2, which plays a central role in apoptosis Wajant and Siegmund (2019). FADD allows the activation of caspase 8 by interacting with its DED domain (death effector domain) Siegmund et al. (2001). TRAF2 is capable of interacting with NIK (NF-*κ*B Inducing Kinase) which belongs to the MEKK family. NIK phosphorylates its target IKK (Inhibitor of *κ*B Kinase) which ultimately leads to the activation of NF-*κ*B. NF-*κ*B produces an anti-apoptotic effect by promoting transcription of cellular inhibitor of apoptosis protein 2 (c-IAP2), which blocks the caspase 8 activation and hence apoptosis (Olmos and Lladó, 2014; Chadwick et al., 2008).

The second messenger functions of sphingolipids allow them to modulate a variety of signaling events in the central nervous system (CNS). In the sphingolipid metabolic pathway, sphingomyelinases (SMase) are of great importance as they catalyze the conversion of sphingomyelin to ceramide. Three types of sphingomyelinases have yet been identified that are categorized based on their optimal pH, i.e., alkaline, acidic, and neutral. In neurons, ceramide is produced by the hydrolysis of sphingomyelin *via* acidic (A-SMase) or neutral (N-SMase) as both of these forms have been identified in the neurons. (Haughey et al., 2010). The activation of both SMases is mediated by the TNF-*α* signaling cascade. The adaptor proteins TRADD and FADD induce the activity of acid SMase (Wiegmann et al., 1999), whereas FAN is found to be associated with the activation of neutral SMase (Ségui et al., 2001). It implicates the role of TNF-*α* mediated cytotoxic effects in the regulation of sphingolipid signaling cascade and hence on neuronal survival.

Ceramide, generated in response to acid sphingomyelinase, activates aspartyl protease cathepsin D responsible for the cleavage and activation of pro-apoptotic Bid (family member Bcl-2). The elevated levels of ceramide cause increased permeability of mitochondria which induces the release of cytochrome C from the mitochondria. It causes the activation of caspases 3 and 9, ultimately leading to apoptosis *via* an intrinsic pathway. On the other hand, ceramide produced *via* neutral sphingomyelinase (nSMase) directly activates two target enzymes, namely, CAPK (ceramide activated protein kinase) and CAPP (ceramide activated protein phosphatase) (Haughey et al., 2010). Both of them are capable of evoking signaling that leads to apoptosis. CAPK phosphorylates raf-1, which enhances its activity towards MEK (MAPK kinase). The MAPK signaling cascade induces activation of ERK cascade, which either results in cell cycle arrest and cell death or contributes to neuronal cell survival depending upon the form of ERK activated. On the other hand, CAPP (comprising of serine-threonine phosphatases PP1 and PP2A) moves the system towards apoptosis by inactivating Akt (Haughey et al., 2010).

### 2.2 Quantitative modeling

#### 2.2.1 Entities concentrations from the microarray analysis

In the current study, we utilized the gene expression values from the microarray data as the initial concentrations for the input entities of the TNF-*α* mediated sphingomyelin pathway model for the purpose of its quantitative analysis. The dataset taken was from the study entitled “Expression data from post mortem Alzheimer’s disease brains” (GEO accession: GSE36980). This data set was selected based on the two selection criteria: it was of *Homo sapiens* origin and was free from any therapy or drug. The data comprised 79 samples from different regions of the brain, out of which 32 samples were from AD patients and 47 from normal individuals. The platform of data was Affymetrix Human Gene 1.0 ST Array[HuGene-1_0-st]. The “maEndToEnd” (http://bioconductor.org/packages/devel/workflows/html/maEndToEnd.html) Bioconductor R package was used for step by step analysis of data (Klaus and Reisenauer, 2016). First, the quality assessment of data was performed by visualizing data using box plot, PCA plot, and Heatmap (Bioconductor R packages (Klaus and Reisenauer, 2016; Gu et al., 2016)). Then linear regression model was fit using the limma R package for obtaining the differentially expressed genes (DEGs). Hence, the use of average gene expressions as the initial values formed a basis for analyzing the effects of variations in normal entities’ concentrations over the whole model.

#### 2.2.2 Quantitative model building

**FIGURE 3:**
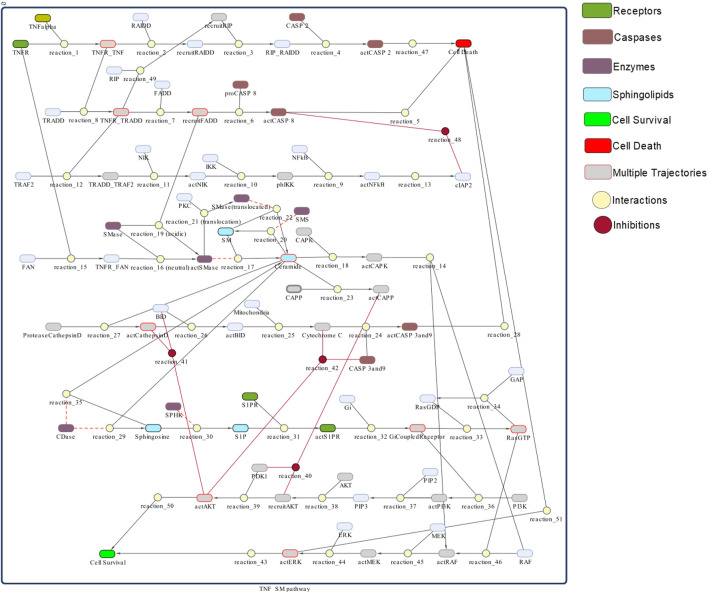
TNF-*α* mediated sphingomyelin signaling pathway model- The whole model is depicted as a network of species (oval) and interactions (circle) connected by lines/edges. Species represent biological entities (receptors, ligands, proteins, enzymes) of the pathway: green color shows receptors, grape color shows caspases, mauve color shows enzymes related to sphingomyelin pathway, light seagreen color shows sphingolipid molecules, bright green color shows cell survival and red color shows cell death. Interactions 1–51 represent processes like transformation, transport, binding/unbinding, activation/inhibition: wine color represents inhibitions. Lines connect species with interactions: red lines show inhibition and dotted lines show read arcs representing reversible effect of enzymes specific to the sphingomyelin pathway. Species with red boundaries have multiple trajectories.

The model of TNF-*α* mediated sphingomyelin signaling pathway is generated in Simbiology (Figure 3). Simbiology is a MATLAB package provided with programmatic tools to simplify modeling, simulation, and analysis of dynamic biological systems. It uses ordinary differential equations and stochastic solvers to simulate and analyse time-course profiles of the model’s entities and parameters (Liu et al., 2010). It provides three building blocks to model dynamic biological systems, i.e., species, compartments, and reactions. Species represent the dynamic states or the concentrations of biological entities like proteins, enzymes, receptors, ligands, or metabolites. Compartments refer to physically isolated environments having a particular set of species, for instance, any particular cell. However, the reactions describe the type of association/interaction among different species. The type of association may represent several biological processes, such as the transformation of molecules, transport of proteins, the binding process of ligands and receptors, or the binding of substrates with enzymes. According to the principle of kinetic law, all interactions are guided by certain kinetics. (All necessary Simbiology model files are publically available at https://github.com/SanamBanaras/Sphingolipid_Signaling_Pathway.git).

Based on mass-balance principle, Simbiology automatically translates the network representation of the model into a set of ordinary differential equations (ODEs), thus, mathematically describing the dynamics of biological systems. If we consider two entities “A” and “B” acting as the reactants that interact to form a product “C,” it can be represented by the reaction:

$$mA\;\;+\;\;nB\;\;\;\overset{k}{\to }\;\;\;C$$ (1)

$$\frac{d\left[C\right]}{dt}\;\;\;\;=\;\;\;\;k\left[A\right]\;\left[B\right]$$ (2)

$$\frac{d\left[A\right]}{dt}\;\;\;\;=\;\;\;\;-k\left[A\right]\;\left[B\right]$$ (3)

$$\frac{d\left[B\right]}{dt}\;\;\;\;=\;\;\;\;-k\left[A\right]\;\left[B\right]$$ (4)

The corresponding system of differential equations for the type of interaction between the entities A and B, represented in reaction 1 and model in Supplementary Figure S2A, is given by Eqs 2–4. In the above set of differential equations, “k” represents the rate constant. The rate of reaction can be defined as the speed of a chemical reaction that is proportional to the change in the concentration of reactant or product per unit time. In terms of product, it is the increase in the concentration of a product per unit time and is represented as d[C]/d[t] for the Eq. 1. In terms of reactant, it is the decrease in the concentration of a reactant per unit time and is represented as d[A]/d[t] or d[B]/d[t] for this equation.

According to the law of mass action, the rate of any chemical reaction is directly proportional to the product of concentrations of the reacting entities (concentrations are represented as “[]”), and each concentration is raised to a power equal to its respective coefficient in the chemical reaction. The coefficients in the chemical equation (reaction), like “m” and “n” (in Eqs 2–4), indicate the number of molecules of the reactants “A” and “B,” respectively. Therefore, for reaction 1, we obtain a corresponding system of differential equations (given by Eqs 2–4) representing the rate of chemical reaction according to the law of mass action. We modeled reaction 1 in Simbiology as shown in the Supplementary Figure S2A. For this model, we set the concentration/amount of the reactant “A” as 20, “B” as 10, and the value of mass action rate constant “k” as 1. The simulation run of the model with given settings, as given in Supplementary Figure S2B, over 5-time units shows the decrease in the concentration of reactants “A” and “B,” while an increase in the concentration of product “C”. The concentration of reactant “A” decreases from 20 to 10, whereas the concentration of reactant B reduces from 10 to 0. However, the amount of product increases from 0 to 10.

The model of TNF-*α* mediated sphingomyelin signaling pathway was constructed as a network of species representing receptors, ligands, enzymes, adaptor proteins or molecules, and interactions representing processes like transformation, transport, activation, inhibition, binding or unbinding; connected by lines (or edges). Each input entity of the model was set to a specific initial value based on its expression profile obtained in the microarray analysis (discussed in Section 3.1). Moreover, all the interactions of model were made to follow mass-action kinetics. Accordingly, the kinetic parameter values for interactions were incorporated in the form of mass action kinetics (kf). These values of mass action kinetics were adjusted between 0 and 0.1 (any suitable value between 0 and 0.1) to attain the homeostatic balanced state of the model. The kinetic values were manipulated due to the lack of kinetic data of the model’s interactions (Koch et al., 2010). The model along with its full description is shown in Figure 3. All interactions are given in the Supplementary Material Section 1. The ordinary differential equations (ODEs) representing rates of model interactions are given in Supplementary Material Section 2 and the adjusted kinetic parameter values for all interactions are given in the Supplementary Material Section 3.

#### 2.2.3 Network analysis

A network refers to a set of connected elements, in a formal process, it can be modeled as mathematical units called graphs.

Definition 1. A directed graph G = (V, E) consists of a finite set of vertices “V” and a finite set of directed edges “E” (Eq. 5). An edge E = (U, V) connects two vertices U and V and is directed from U to V.

E⊆V×V (5)

Different types of biological entities and interactions of the model possess a high significance in systems biology. Network analysis approaches having non-biological bases can be effectively implemented on large and complex biological networks to search pivotal components. One such measure is the centrality analysis of pathway elements, which allows the ranking and identification of significant entities of the model based on their position. For instance, the functional importance of highly connected vertices in protein networks is evident as their removal or deletion can cause lethality (Valente et al., 2008). The two centrality measures, i.e., betweenness centrality and closeness centrality were applied to the model, which quantified the significance of certain entities among others. Betweenness centrality determines a node’s centrality by measuring shortest paths from all vertices to all other vertices of the graph that pass through that node. On the other hand, closeness centrality applied to strongly connected graphs utilizes information about length of the shortest paths within a network. The closeness centrality can be found by taking the reciprocal of the sum of minimal distances of a vertex to all other vertices.Definition 2. Formally, if we denote by ${\sigma \;}^{x^{\prime }x^{\prime \prime }}$ the number of shortest paths from x’ to x” and by ${\sigma \;}^{x^{\prime }x^{\prime \prime }}$ (x) the number of these shortest paths that go through node x, then the betweenness centrality C_*B*_(x) for given node x is defined as

CBx=∑x′,x″∈Vx′≠x≠x″σx′x″xσx′x″ (6)

Definition 3. The closeness centrality C_*C*_(x) of a node x in a graph G = (V, E, W) is defined as the inverse of the sum of the shortest path lengths from this node to every other node in the graph.

$$CC\left(x\right)={\left(\underset{x^{\prime }\in V}{\sum }sG\left(x,x^{\prime }\right)\right)}^{-1}$$ (7)

#### 2.2.4 Simulation of the model

Simbiology can simulate the dynamic behavior of the model *via* converting interactions and rate parameters into a set of ordinary differential equations. Different ODE solvers (ODE 15s, ODE 23t) are utilized to compute solutions for ODEs at different times during the simulation. An exemplar simulation run for the model shown in Supplementary Figure S2A is given in the Supplementary Figure S2B. The simulation results show the gradual decrease in reactants A and B concentrations while an increase in product C concentration with mass action kinetic equal to 1. We performed simulation of the generated model of TNF-*α* mediated sphingomyelin signaling pathway to track precisely the minor details of changes in entities’ concentrations over time (from 0 to 100) till the attainment of the homeostatic state. The relative time unit (RTU) was considered as the unit of time during simulation. The simulation plots mimicked the gradual process of change in the concentrations (quantities) of the entities with respect to time.

#### 2.2.5 Scanning of the model components

The scanning allows determining the trend or changing behavior of the system entities (in the form of varying concentrations) over an alternating range of a particular component of the model (entity or a parameter). In the current study, we conducted two scanning procedures on the model, i.e., Parameter scanning and Entity scanning. The parameter scanning determined the trend or behavior of a model entity (trend of varying concentration) in response to an increase in parameter (kinetic) value of a particular interaction within a specified range. On the other hand, entity scanning depicted an entity’s response (trend of changing concentration) towards alternating concentration of a particular model entity over a specified range. In the current study, parameter scanning was performed to observe and determine the effect of alternating kinetics on neuronal homeostasis. Moreover, the adjusted kinetic values were compared with the known kinetic parameter values for some of the interactions (represented by Eqs 8–16 of TNF-*α* signaling cascade (Table 1) to determine and quantify the difference in their impact on neuronal homeostasis. Table 1 shows the known kinetic rates for some interactions of TNF-*α* signaling cascade along with their tolerable ranges of variations. Moreover, the comparison between the adjusted and literature kinetic values enabled to determine the likelihood of occurrence of adjusted values for the interactions with known kinetic parameter values within tolerable ranges of variation according to Table 1.

**TABLE 1:** Interactions with their Kinetic Parameters (concentration in μM and time in seconds) from Literature along with corresponding range of variation.

interactions	Kinetics (μ*M* ^−1^ *s* ^−1^)	Range of variation	References
TNFR + TNF-*α* → TNF-*α*_TNFR	0.185	0.00925–2.775	Cho et al. (2003)
TRADD + TNFR_TNF-*α* → TNFR_TRADD	0.185	0.00925–2.775	Cho et al. (2003)
TRAF2+TNFR_TRADD → TRADD_TRAF2	0.185	0.00925–2.775	Cho et al. (2003)
RIP1+TNFR_TRADD → TRADD_RIP1	0.185	-	Cho et al. (2003)
FADD + TRADD → TRADD_FADD	0.185	0.00925–2.775	Cho et al. (2003)
FADD + CASP8 → CASP8*	0.185	-	Cho et al. (2003)
IKK + NF*κ*B → IKK_NF*κ*B	0.185	-	Cho et al. (2003)
RIP1+ CASP8 → RIP1_CASP8	0.0925	0.0462–0.1387	Cho et al. (2003)


$$TNF-\alpha \;+\;TNFR\;\;\overset{k=0.185}{\to }\;\;TNF-\alpha \text{\_}TNFR$$ (8)

$$TNF-\alpha \text{\_}TNFR\;+\;RAIDD\;\;\overset{k=0.1\left(adjusted\right)}{\to }\;\;recruitRAIDD$$ (9)

$$TNF-\alpha \text{\_}TNFR\;+\;TRADD\;\;\overset{k=0.185}{\to }\;\;TNFR\text{\_}TRADD$$ (10)

$$TNFR\text{\_}TRADD\;+\;FADD\;\;\overset{k=0.185}{\to }\;\;recruitFADD$$ (11)

$$NFkB\;+\;phIKK\;\;\overset{k=0.185}{\to }\;\;actNFkB$$ (12)

$$TNFR\text{\_}TRADD\;+\;TRAF2\;\;\overset{k=0.185}{\to }\;\;TRADD\text{\_}TRAF2$$ (13)

$$TNFR\;+\;FAN\;\;\overset{k=0.1\left(adjusted\right)}{\to }\;\;TNFR\text{\_}FAN$$ (14)

$$S1P\;+\;S1PR\;\;\overset{k=0.1\left(adjusted\right)}{\to }\;\;actS1PR$$ (15)

$$AKT\;+\;PIP3\;\;\overset{k=0.1\left(adjusted\right)}{\to }\;\;recruitAKT$$ (16)

#### 2.2.6 Sensitivity analysis

Sensitivity analysis determines the sensitivity or susceptibility of species (entities) or parameters (kinetics) in a model to a specific condition defined by a species or parameter. In Simbiology, the sensitivity analysis is supported by ODE solvers and is categorized into two classes, i.e., local sensitivity analysis and global sensitivity analysis. Local sensitivity is a one at time (OAT) technique that analyzes the effect of a single parameter while keeping all the others fixed. On the other hand, global sensitivity calculation involves the manipulation of collective parameters to explore the design space. Furthermore, the sensitivity analysis can be conducted in two ways, i.e., species (entities) versus parameters (kinetics) or species (entities) versus species (entities). We performed both forms of sensitivity analysis on the TNF-*α* mediated sphingomyelin pathway model. We took all the reaction (interaction) parameters as inputs and entities as outputs to determine the sensitivities of entities in response to reaction (interaction) parameters. Likewise, we determined the influence of the model entities on other entities by selecting all entities as both the input and output.

#### 2.2.7 Intervention of drugs in the model and their response

Different neuropathologies including immune disease, trauma, and inflammation may lead to rapid and irreversible neurological damage. However, various pharmacological treatments have been formulated and applied for the aforementioned neurological disorders and many other diseases. Etanercept, a TNF-*α* antagonist is used as a TNF-*α* blocker to minimize the neurological damage mediated by TNF-dependent processes (Kondziella and Waldemar, 2017). Nivocasan is an irreversible inhibitor of caspase 1, caspase 8, and caspase 9 (Kudelova et al., 2015), and is recommended for the apoptosis-mediated liver injury (Adriaenssens, 2017). Scyphostatin inhibits the neutral SMase (Pitsinos et al., 2003) and is implicated in lowering the apoptotic effects by decreasing ceramide production.

Pharmacokinetic modeling in Simbiology offers a mathematical approach for predicting the effectiveness and efficacy of drug dosage on the model over time. Using this approach, we assessed the efficacy of three drugs, namely, Etanercept, Nivocasan, and Scyphostatin on TNF-*α* mediated sphingomyelin signaling pathway model. These drugs were assimilated as species in our model, and each drug was analyzed individually. The combined analysis of each drug involving simulation (described in Section 2.2.4), scanning (described in Section 2.2.5), and dosing schedule enabled to study a comprehensive model’s response towards it. The “dosing” allows to assimilate the quantity of a specific species (taken as a drug) in model during simulation. In the current study, we adapted the repeat dose strategy to analyze the influence of three mentioned drugs on neuronal apoptosis and survival.

The information of relevant drugs or bioactive molecules having drug-like properties can be obtained using the CHEMBL database (https://www.ebi.ac.uk/chembl/). For the current model, the targets were selected based on two factors, i.e., availability of FDA-approved drugs and the potency of both drugs and targets to lower neuronal apoptosis. The details of drugs against which the model’s response was studied is given in Table 2, indicating the name of the drugs along with their targets and mode of mechanism. The model showing drugs with their corresponding site of action is given in Supplementary Figure S3.

**TABLE 2:** Drugs for TNF-*α* mediated sphingomyelin signaling pathway from CHEMBL (https://www.ebi.ac.uk/chembl/).

Drug Name	Chembl ID	Target	Mechanism	IC50	References
Etanercept	Chembl1201572	TNF-*α*	TNF-*α* inhibition	10^–12^	Tobinick (2016)
Nivocasan	Chembl2105721	CASP 8, CASP 9	CASP 8,9 inhibition	-	-
Scyphostatin	Chembl418376	NSMase	Inhibitory activity against NSMase	10^–6^	Pitsinos et al. (2003)

## 3 Results

We have studied the dynamics of the TNF-*α* mediated sphingomyelin signaling pathway in neurons not only under normal circumstances but in a diseased scenario as well. Microglia and astrocytes release a large amount of TNF-*α* to recompense various pathological afflictions like AD and PD (Smith et al., 2012). The difference in the levels of TNF-*α* causes an imbalance of TNF-*α* regulated signaling cascades having direct or indirect linkage. It eventually alters the equilibrium ratio of neuronal survival and apoptosis. The quantitative modeling of TNF-*α* modulated mechanisms using the Systems Biology approach rendered a distinctive merging of quantitative experimental data with dynamic mathematical modeling, thus providing deeper insights to the model. The quantitative analysis of the model helps to determine the behavior of TNFR-associated adaptor proteins, along with their impact on sphingolipids and enzymes. Moreover, it enables to determine the efficacy and potency of a range of drugs therapeutically active for specific targets in the model by scrutinizing the model response towards different drugs’ dosages.

### 3.1 Quantitative model building and microarray analysis

The model of TNF-*α* mediated sphingomyelin and downstream signaling pathways was built in Simbiology (Figure 3) previously described in Section 2.2.2. An estimation of initial concentrations for input entities of the model from microarray gene expression data was imperative for envisioning their normal profile in the brain. The names of input model receptors, adaptor proteins and enzymes along with their gene ID’s and average gene expression are given in Table 3. Moreover, the values of three entities, i.e., SM, CAPK, and mitochondria were assumed due to the lack of availability of their expression data. Therefore, we calculated the values of these entities by taking an average of the gene expression values of remaining model entities having known gene expression values.

**TABLE 3:** Average gene expression values of Model Input Entities obtained from the analysis of Microarray Dataset (GSE36980).

Gene ID	Description	Average gene expression
7897877	TNFR	8.243984468
8177983	TNF-*α*	6.536676383
7957560	RAIDD	7.788116383
7941927	RIP	8.26486
8136869	CASP 2	8.382271277
8001938	TRADD	8.054042766
7942168	FADD	8.231118085
8047419	CASP 8 (pro CASP 8)	6.960822128
8159476	TRAF2	7.739208936
8016194	NIK	7.416519362
7935707	IKK	8.132140851
8096635	NFkB	8.291935532
7943424	cIAP2	8.945781277
8150928	FAN	9.14069
7938100	acid_SMase	8.81918234
8121418	neutral_SMase	8.046917021
8034762	PKC	11.0
	SM*	8.0
	CAPK*	8.0
8114158	CAPP	10.96888511
7945666	Cathepsin D	11.5453383
8074261	BID	8.937911064
8103922	CASP3	7.36558
7912646	CASP9	8.748945106
8033151	CDase	6.900429787
8030078	SPHK	8.642491915
7903393	S1PR1	8.332580213
8133860	Gi	9.610689149
7945436	RasGDP	9.31107383
7947681	GAP	10.40175149
8084016	PI3K	9.115858298
8164328	PIP2	7.307359
7925531	AKT	11.38724894
8046408	PDK1	8.90167
8085164	RAF	9.895470426
7984319	MEK	10.74989787
8074791	ERK	12.18609574
	Mitochondria	8.0
8166402	SMS	9.51

### 3.2 Network analysis

**FIGURE 4:**
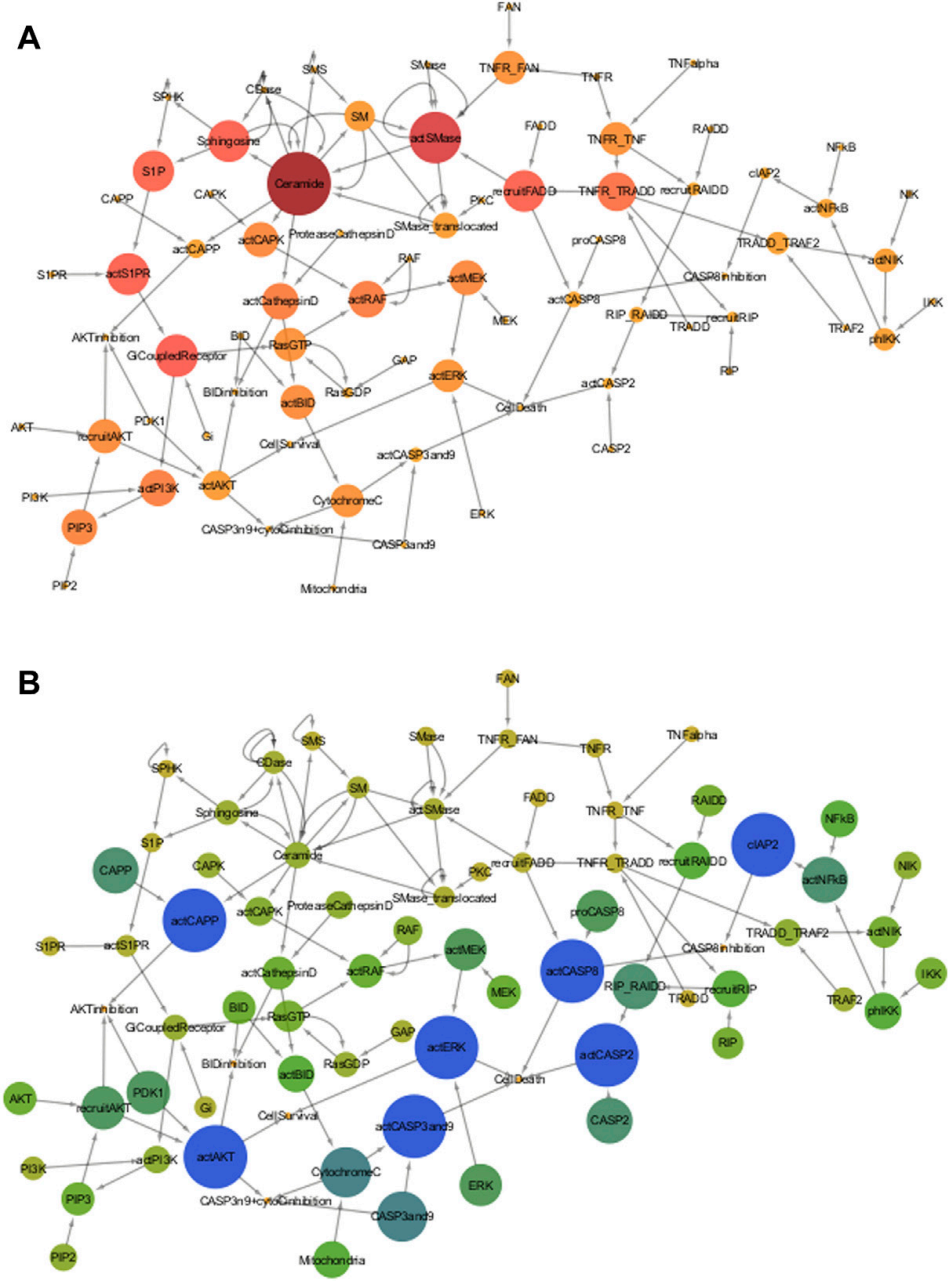
**(A)** Betweenness Centrality of TNF-*α* mediated sphingomyelin signaling pathway- Betweenness Centrality of a node represents the number of shortest paths between other pair of nodes that pass through that node. In this figure, the betweenness centralities of the pathway entities are represented by the color and size of the nodes, varying from dark wine to amber yellow and large to small, respectively. Nodes with dark wine color and large size have high betweenness centrality, whereas nodes with light golden color and small size have low betweenness centrality. Color of intermediate nodes vary from punch pink to amber. **(B)** Closeness Centrality of TNF-*α* mediated sphingomyelin signaling pathway- Closeness Centrality of nodes determines their average distance to all other nodes. The closeness centralities of the pathway entities are represented by the color and size of the nodes, varying from dark blue to yellow and large to small respectively. Dark blue nodes with large sizes have high closeness centrality, whereas yellow color nodes having small sizes have low betweenness centrality. Color of intermediate nodes vary from pine green to light green.

Model of the TNF-*α* mediated sphingomyelin and associated downstream signaling pathways presented a viable medium for the network analysis (Figure 4). The values for betweenness centrality and closeness centrality of all the entities of model are given in the Supplementary Material Section 4 and 5, respectively. The betweenness centrality results show that the ceramide has the highest value (0.078) among all other entities of the model (Figure 4A). The ranking of the entities betweenness centralities values can be explained by the number and type of interactions with other entities. The ceramide has a total of 13 interactions with other entities, among them, six interactions are incoming while seven are outgoing ones. Likewise, Buccoliero and Futerman (2003) presented number of evidences indicating the central role of ceramide in the regulation of neuronal survival and apoptosis (Buccoliero and Futerman, 2003). The rate of dendritic growth is reduced (due to lack of ceramide) in cell line of hippocampal neurons *via* the application of fumonisin B which causes an inhibition of ceramide synthesis (Schwarz and Futerman, 1998). Moreover, Teitsdottir et al. (2021) reported high levels of ceramide as a measure of increased AD pathology and their direct impact on neuronal apoptosis during AD pathogenesis *via* cerebrospinal fluid (CSF) lipidomic analysis (Teitsdottir et al., 2021). The second highest betweenness value is of actSMase (0.051) and has a total of seven interactions (five incoming, two outgoing). The third-largest value is of GiCoupledReceptor (0.031), with a total of 4 interactions (two incoming, two outgoing). The forth is actS1PR (0.03), with a total of three interactions (two incoming, one outgoing). The next value is for Sphingosine (0.029), with a total of six interactions (two incoming, four outgoing). The sixth value is of recruitFADD (0.029), with a total of 4 interactions (two incoming, two outgoing). The seventh value is S1P (0.028), with a total of 3 interactions (two incoming, one outgoing). The eighth value is of TNFR/TRADD (0.022), with a total of five interactions (two incoming, three outgoing). The betweenness values of entities decrease with the number of incoming interactions irrespective of the number of outgoing interactions. Later in the Section 3.3.3.2, the effects of the scanning of these entities on neuronal survival and apoptosis.

On the other hand, entities like actCASP2, actCaspase 8, cIAP2, actCAPP, actCaspase 3 and 9, act, and showed the highest closeness centralities with value 1. The highest values suggest their significance because of having a minimum distance from all other entities in the model. Due to this reason, they can serve as the hub entities of the model. Cytochrome C and Caspase 3 and 9 have the second-highest values of 0.75. The high closeness values of caspases signify their involvement in non-apoptotic processes during neuronal development or diseased state (Mukherjee and Williams, 2017). The closeness centralities of all entities is given in Figure 4B. The effects of scanning of these entities on the neuronal survival and apoptosis later in the Section 3.3.3.3.

### 3.3 Quantitative analysis of TNF-*α* mediated sphingomyelin signaling pathway model

The comprehensive set of quantitative data in the form of initial concentrations as determined from microarray technique and adjusted reaction (interaction) rates rendered quantitative study and analysis of TNF-*α* mediated sphingomyelin pathway model. The values (or concentrations) for input entities of model were the average gene expressions of normal/healthy conditions. The kinetics parameters of the model’s interactions were adjusted between the values 0 to 0.1 with an aim to attain the balanced state of the model in normal/healthy condition. This balanced state of model showed the uniformity in neuronal survival and apoptotic mechanisms, thus, depicting the normal and healthy neuronal conditions. The depiction of a stable neuronal condition was necessary to analyze the effect of any variation (in entity concentration or kinetic parameter) in the diseased state later in our study. The kinetic parameter values of all interactions of the model are provided in the Supplementary Material Section 3.

#### 3.3.1 Simulation of the model

**FIGURE 5:**
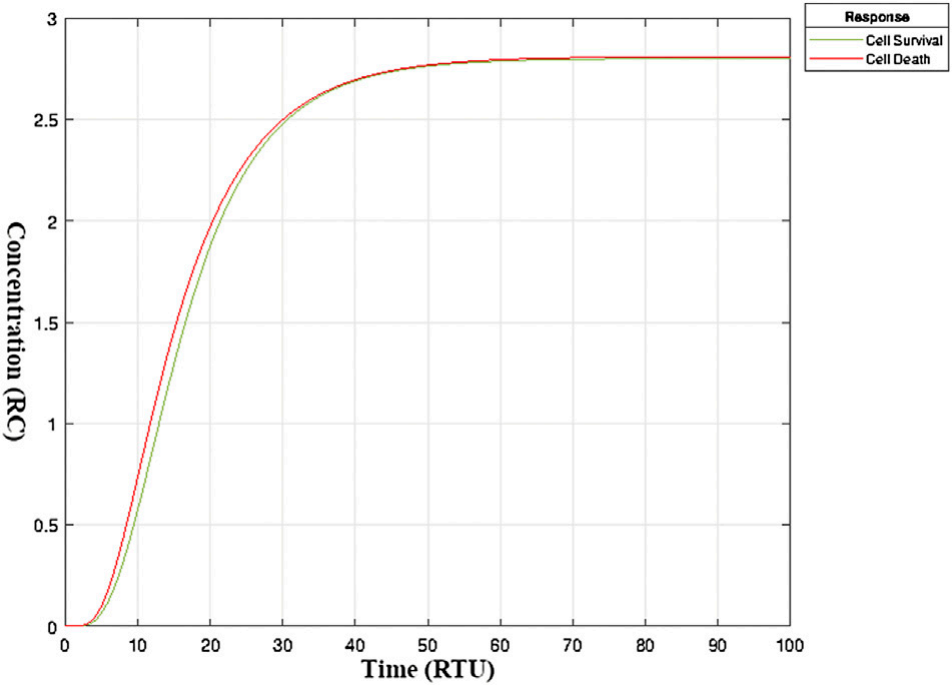
Balanced State of neuronal survival and neuronal apoptosis in mature neurons- Balanced State of neuronal survival and neuronal apoptosis in TNF-*α* mediated sphingomyelin signaling pathway model under normal circumstances. The *y*-axis represents the extent or level (in relative concentration) of neuronal survival and apoptosis. The *x*-axis represents relative time. The two curves, i.e., green and red, are the outcomes of the physiological processes in neurons leading to neuronal survival and death, respectively. The interactive network of signaling pathways keeps neuronal survival and apoptosis in balance (Kole et al., 2013). A shift in one or more of these signaling pathways can alter the fate of a neuron resulting in neuronal death or continued survival (Morrison et al., 2003).

The simulation run of a model in Simbiology enabled us to track precisely the minor details of even minute changes in entities concentrations over time. Figure 5 shows the hypothesized stable (balanced) state of TNF-*α* mediated sphingomyelin and related signaling pathways exhibited by fully developed post-mitotic neurons under normal circumstances. It can be noticed that both survival and apoptotic mechanisms (in terms of relative concentration (RC)) function in harmony to keep the system in balance. This is the neuronal physiological state with no influence on the neuronal cell count. The *y*-axis in Figure 5 indicates the extent of neuronal survival and apoptotic curves till the attainment of an equilibrium state.

As evident from Supplementary Figure S4A, TNFR receptor takes approximately 7-8 time units to be occupied by TNF-*α* (having amount 3.4) and FAN (4.74). Also, the activation of caspase 2 (actCASP 2) is at its maximum (0.071) at 12–14 time units, and it gradually drops to a minimum of 0.015 in approximately 37-time units.

The formation of TNFR_TRADD reaches the peak value of 0.56 in 3 time units and takes further 6 time units to drop. The recruitment of FADD (recruitFADD) is at the highest with the value 0.0215 in 2 time units and drops to a minimum in 6 time units. Maximum activation of caspase 8 (actCASP 8) takes place in approximately 6 time units (0.038) and then drops to the minimum (0.006) in about 25 time units. The formation of cIAP2 takes place after 3 time units with a rate of 0.1. After that, it reaches its maximum value of 1.4 in nearly 60 time units and then becomes constant. The rate of cIAP2 formation is very high till the attainment of value 1 in the first 20 time units. (Supplementary Figure S4B) The activation of Ceramide activated protein phosphatase (actCAPP) reaches the maximum value of 2.2 in 8–10 time units. The constant amount of actCAPP shows its minimum utility in the subsequent interaction possibly because of the small rate. The ceramide production immediately reaches the maximum of 0.52 after 2 units and drops to a minimum of 0.05 right after 10 time units. Similarly, activation of SMase (actSMase) takes place immediately and reaches the maximum 1.48 at 2.5 time units for the adjusted rate and drops to a minimum of 0.03 in approximately 10 time units as given in Supplementary Figure S4C.

The release of cytochrome C from mitochondria reaches the maximum (0.235) with 0.1 kinetics in nearly 6 time units. The amount of caspases 3 and 9 starts to decline after 2 time units, and gradually drops to a constant value of 12.7 in 18 time units. The full activation of caspases 3 and 9 takes 9 time units to reach the maximum value of 1.35 with a rate of 0.1 and then drops to the minimum in 65–70 time units. (Supplementary Figure S4D). Formation of sphingosine reaches the maximum value of 0.34 in 3 time units and drops to a minimum in 10–15 time units. The production of sphingosine-1 phosphate (S1P) attains the peak value of 0.32 at 4.5 time units with a rate of 0.1 and takes 15–20 time units to drop. The activation of S1PR reaches the peak value of 0.245 in approximately 6.5 time units with a reaction rate of 0.1 and then drops to minimum in 15–20 time units. The triggering of the Gi Coupled receptor takes place after 1 time unit and reaches a maximum value of 0.12 in nearly seven time units with the reaction rate of 0.1. Moreover, it takes 18–20 time units to reach the minimum (Supplementary Figure S4E) As indicated in Supplementary Figure S4F, the activation of AKT (actAKT) takes place after 3 time units and reaches to the maximum value of 0.33 with the reaction rate of 0.1. However, the activation of ERK (actERK) takes place after 1 time unit and reaches the maximum value of 1.2 in 11–12 time units.

#### 3.3.2 Parameter scanning

**FIGURE 6:**
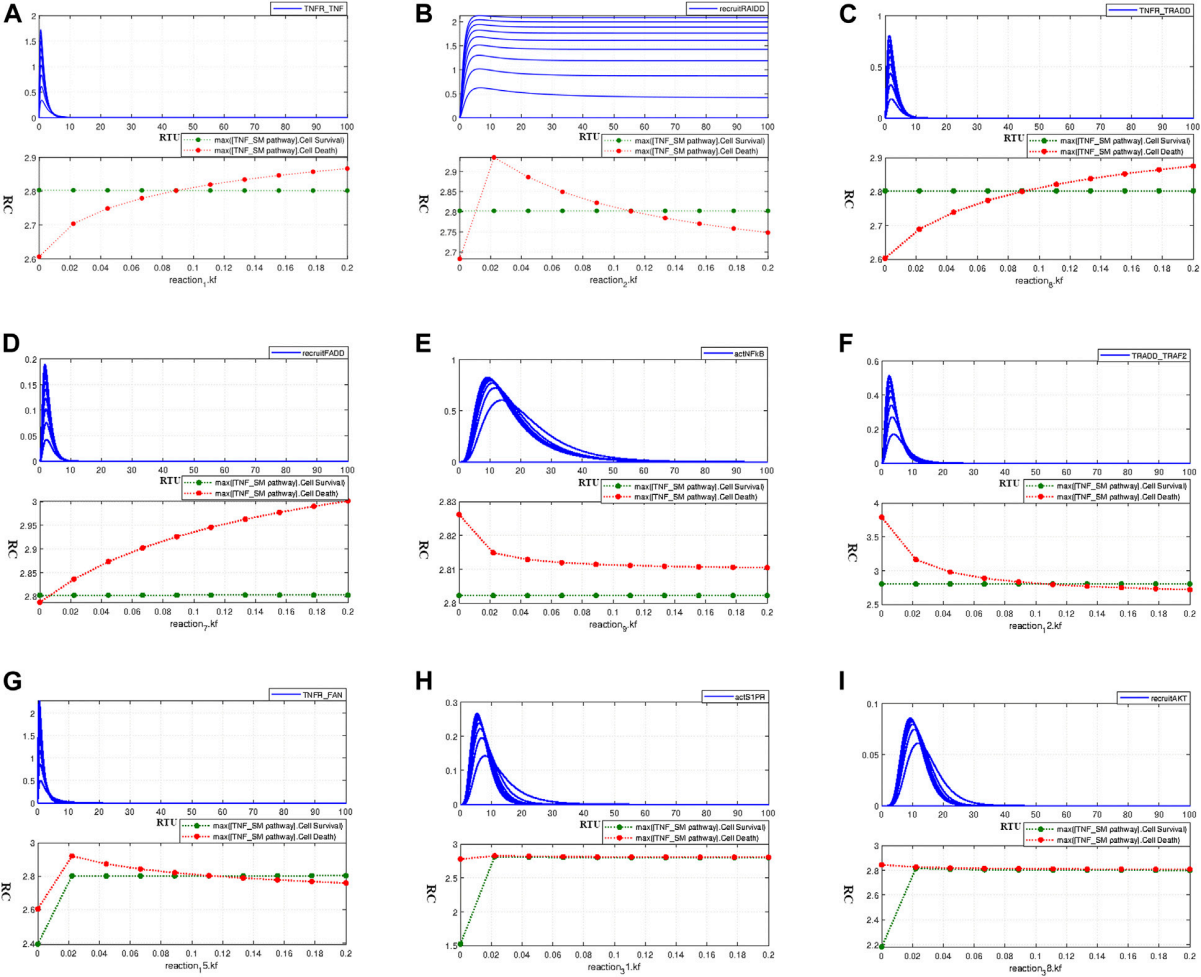
Paramater Scanning of TNF-*α* mediated Sphingolipid Signaling Pathway model interactions with known kinetic parameters from the literature to determine their tolerable range of variations and to study their effect on neuronal survival and death- **(A)** Paramater scanning for interaction 1 (Eq. 8) representing TNFR_TNF*α* formation. **(B)** Paramater scanning for interaction 2 (Eq. 9) representing recruitment of RAIDD. **(C)** Paramater scanning for interaction 8 (Eq. 10) representing formation of TNF_TRADD. **(D)** Paramater scanning for interaction 7 (Eq. 11) representing recruitment of FADD. **(E)** Paramater scanning for interaction 9 (Eq. 12) representing NF*κ*B activation. **(F)** Paramater scanning for interaction 12 (Eq. 13) representing TRADD_TRAF2 comlex formation. **(G)** Paramater scanning for interaction 15 (Eq. 14) representing TNFR_FAN complex formation.**(H)** Paramater scanning for interaction 31 (Eq. 15) representing activation of S1PR. **(I)** Paramater scanning for interaction 38 (Eq. 16) representing recruitment of AKT.

The interaction 1 of the model is given by Eq. 8 which indicates the binding of TNF-*α* with TNFR receptor. Parameter scanning graph for interaction 1 (Eq. 8) given in Figure 6A shows a balance between cell survival (green) and cell death (red) at the point of their overlap; where the rate is approximately close to 0.1. It can be traced out that the kinetic parameter value of 0.185 (as give in Table 1) shows only a slight (about 0.04) relative increase in cell apoptosis. Besides, the adjusted rate value 0.1 for the current interaction is within the bounds of the range provided in Table 1. Figure 6A shows the effect of increasing kinetic parameter on TNFR_TNF-*α* formation. The interaction 2 in the model represented by Eq. 9 represents the recruitment of RAIDD after TNFR activation. We adjusted the kinetic parameter value for this interaction as 0.1. Figure 6B shows the intersecting point of two curves representing neuronal survival and apoptosis at a kinetic parameter value of 0.11, which implies the balanced state of the system.

The interaction 8 in model representing the formation of the TNFR_TRADD complex is depicted in Eq. 10. In the current model, we adjusted the kinetic parameter value for this interaction as 0.1. The corresponding parameter scanning results are given in Figure 6C. The junction point of neuronal survival and apoptotic plots at the value 0.1 shows the balanced state of the model. This adjusted parameter value has difference of 0.08 units from the value 0.185 as given in Table 1, and it lies within the range of variation provided by Cho et al., (2003), causing a very slight relative difference of 0.05 units among neuronal apoptosis and survival. The interaction 7 in the model represents the recruitment of FADD which follows the TNFR_TRADD complex formation and is represented by Eq. 11. We adjusted the kinetic parameter value for this interaction as 0.01. Parameter scanning results for this interaction are given by Figure 6D which shows the coinciding point of the neuronal survival and apoptotic curves almost at the adjusted value 0.01. This adjusted value lies within the tolerable range of parameter variation given in Table 1, the curves in Figure 6D shows the relative difference of approximately 0.2 units between neuronal apoptosis and survival at a kinetic parameter value of 0.185 from literature.

As discussed earlier, the activation signal for NF*κ*B leads to the phosphorylation of IKK, which sequentially triggers the activation of NF*κ*B. The interaction 9 in the model represents the NF*κ*B activation *via* phIKK and is given by Eq. 12. The adjusted rate value for this interaction is 0.1. The parameter scanning results for this interaction, as given in Figure 6E, show exceptional behaviour as both survival and apoptotic curves move in parallel without coinciding at or near to the adjusted value of 0.1 or the literature value of 0.185 as provided in Table 1.

The TRADD_TRAF2 complex formation (interaction 12 in the model), as represented by Eq. 13, takes place right after the recruitment of adaptor protein TRADD. The adjusted parameter value used for this interaction in the current model is 0.1, whereas the kinetic parameter value obtained from literature is 0.185 (Table 1). The corresponding parameter scanning results are given in Figure 6F. Both the survival and death curves coincide at the adjusted value, which is within the bounds of the range provided by Table 1. Additionally, we can observe a very slight relative difference between survival and apoptotic curves at the kinetic value of 0.185 Cho et al. (2003). Eq. 14 represents the association of FAN with TNFR (which is the interaction 15 in the model). The value of kinetic parameter for the current interaction is adjusted as 0.1. Parameter scanning results in Figure 6G show that the intersecting point of the survival and death curve is nearly around the adjusted value of 0.1. Moreover, the parameter range from 0.02 to 0.2 has no obvious impact on the relative difference between the survival and apoptotic curves.

The binding of S1P with S1PR is represented by Eq. 15 (the interaction 31 in the model). The value of kinetic parameter for this ligand-receptor binding was taken as 0.1. The parameter scanning results in Figure 6H indicate that the parameter range 0.02–0.2 does not even slightly affect the cell homeostatic state, and the system remains in balance. The recruitment of AKT *via* PIP3 (the interaction 38 in the model) is represented by Eq. 16 with the adjusted kinetic parameter value of 0.1 in model. It is evident from the parameter scanning results given in Figure 6I that the parameter range 0.02–0.2 has zero influence on neuronal survival and apoptosis. It can be inferred from parameter scanning results for interactions 1 (Eq. 8), 2 (Eq. 9), 7 (Eq.11), 8 (Eq. 10), 12 (Eq. 13), 15 (Eq. 14), 31 (Eq. 15), and 38 (Eq. 16) that the intersection points of all curves almost coincides with the adjusted parameter kinetics depicting the homeostatic state of the model. Furthermore, except for interaction 9 (Eq. 12), scanning graphs of kinetic parameters over the range 0–0.2 for interactions 1 (Eq. 8), 7 (Eq. 11), 8 (Eq. 10), and 12 (Eq. 13) show that certain values of kinetic parameters above and below the formulated kinetics are bearable, and do not produce profound effects on neuronal survival and neuronal apoptosis. Their comparison with literature kinetic parameter values (Cho et al., 2003) shows that they are in accordance and within the bounds of tolerable ranges presented in literature and causes no drastic shift on the homeostatic state of model.

#### 3.3.3 Scanning of entities

TNF-*α* mediated sphingomyelin signaling model possess various traverses or crosstalks indicating their pivotal role in cell signaling. The presence of multiple trajectories as indicated in Figure 3 signifies the importance of TNFR_TNF, TNFR_TRADD, recruited FADD, ceramide, Gi-coupled receptor, actAKT and actERK in the whole model. The quantitative scanning of the various proteins/enzymes serving as the crosstalks and having high centrality measures provides more insights in understanding their behavior towards different perturbations.

##### 3.3.3.1 Scanning of the input entities

It is evident from the scanning results in Supplementary Figure S5A that cell survival remains unaffected with an increase in TNF-*α* concentration, whereas we observe a gradual increase in neuronal death. The adjusted kinetics incorporated in the model shows the exact balance between cell death and cell survival at a concentration of 6.5. The system moves towards apoptosis with a maximum relative difference of 0.2 units in response to a continuous increase in TNF-*α* concentration, as in the case of the diseased scenario. The elevated TNF-*α* levels are reportedly associated with the secondary neuronal damage in traumatic brain injury (TBI) (Baratz et al., 2015). Baratz et al., (2015) studied the effect of TNF-*α* synthesis inhibitor 3,6′-dithiothalidomide on mice subjected to mild TBI. 3,6′-dithiothalidomide ameliorated the neuronal loss, cognitive impairments and elevations in astrocyte number due in response to mild TBI *via* preventing mTBI-induced TNF-*α* elevation Baratz et al., (2015).

TRADD shows the exact balance between cell survival and cell death at an adjusted kinetic parameter of 0.1 with an initial concentration of 8 as given by scanning results in Supplementary Figure S5B. The increased stimulation of TRADD in response to TNFR activation slightly moves the system towards apoptosis, whereas the survival mechanisms remain unaffected. The experimental studies show an upregulation of TRADD associated with the cell death machinery in AD patients (Zhao et al., 2003). The scanning result for the FAN is given by Figure Supplementary Figure S5C. Intersecting point of the graph shows balance in survival and apoptotic mechanisms of the neuron at the normal levels of FAN incorporated in the model. Also, it can be observed that increased concentrations of FAN slightly shifts the equilibrium state of the model by decreasing neuronal apoptosis.

##### 3.3.3.2 Scanning of entities with high betweenness centrality

The scanning results of ceramide over the range of values from 0 to 50 are given in Supplementary Figure S5C. The elevation in ceramide levels increases both neuronal survival and apoptosis up to the specified value with the 3-fold relative difference. Toman et al. (2000) examined the dual role of ceramide in promoting neuronal cell death and apoptosis (Toman et al., 2000). However, the curve for neuronal survival is slightly higher when compared to the apoptotic curve. It can be justified by the fact that increased levels of ceramide result in an increased activity of CAPK and CDase enzymes, which compels the system towards survival mechanisms. The scanning results of Gi-coupled Receptor over range 0–50 are given in Figure Supplementary Figure S6B. Neuronal apoptosis is not affected by increased activation of Gi-Coupled Receptors. On the other hand, the neuronal survival rate increases with a maximum relative difference of about 12 folds among survival and death curves.

The scanning results for recruited FADD are given by Supplementary Figure S6C. An increase in recruitFADD concentration leads to increased neuronal apoptosis up to the specified value of 25 with the relative difference of about 6-folds between survival and apoptotic curves. However, the neuronal survival remains unaffected by the recruitFADD concentration. Supplementary Figure S6D shows the scanning results of TNFR_TRADD. It can be observed that the neuronal survival remains unaffected by an increase in TNFR_TRADD concentration from 0 to 50. However, the neuronal apoptosis increases to the maximum with the relative difference of about 3-folds among survival and death curves. Scanning results for actSMase, actS1PR, Sphingosine, and S1P are given in Supplementary Figure S6E–H respectively. The increased levels of actSMase cause a very slight increase in neuronal survival and apoptotic mechanisms with a negligible relative difference of about 0.02 folds. On the other hand, increased levels of actS1PR, sphingosine, and S1P result increase survival mechanism, without affecting neuronal apoptosis, with the relative difference of about 5-6 folds.

##### 3.3.3.3 Scanning of entities with high closeness centrality

The scanning of actAKT over range 0–50 was performed (Supplementary Figure S7A shows). Neuronal apoptosis remains unaffected from increased levels of actAKT. However, an increase in neuronal survival can be observed with a large relative difference of about 50-folds at actAKT value 50. Endo et al. (2006) reported an upregulation in Akt signaling during ishemia and other neurodegenerative disorders to mediate neuronal survival (Endo et al., 2006). The scanning results of actERK as indicated in Supplementary Figure S7B shows no influence of actERK concentration on neuronal apoptosis. However, the impact of actERK on neuronal survival is nearly similar to that caused by actAKT. The increase in actERK value up to 50 leads to increased neuronal survival with the relative difference of about 50-folds.

The scanning results of actCASP2, actCASP8, cIAP2, and actCAPP are given in Figure Supplementary Figure S7C–F respectively. The increased levels of actCASP2 and actCASP8 result in increased apoptosis with a large relative difference; without influencing survival rate. The increased levels of cIAP2 cause a slight lowering of neuronal apoptosis with the negligible relative difference of about 0.02 folds. The increased concentration of actCAPP only lowers the survival curve due to its inhibitory effect with a very slight relative difference of about 0.4 folds. The scanning results for actCASP 3and9, Cytochrome C, RIP_RAIDD and actNF*κ*B are given in Supplementary Figure S7G–J respectively. Increased levels of actCASP 3and9 profoundly affect neuronal apoptosis with a relative difference of about 60-folds. The increased levels of cytochrome C and RIP_RAIDD raise neuronal apoptosis with the relative difference of 15 and 10 folds, respectively. The increased activation of NFkB lowers neuronal apoptosis with a negligible relative difference.

Supplementary Figure S6B, Supplementary Figure S7A, and Supplementary Figure S7B show increasing trends in cell growth with increased activation of Gi-Coupled Receptor, AKT, and ERK respectively. On the other hand, the high levels of recruited FADD (Supplementary Figure S6C) and TNFR_TRADD (Supplementary Figure S6D) having betweenness centrality of 0.02 enhance neuronal apoptosis as evident from the literature. However, despite its suggested role in apoptosis, the increasing levels of ceramide intensify both cell growth and cell death (Supplementary Figure S6A). This contradiction can be explained by the increased activity of CAPK and ceramidase due to elevated ceramide levels, which are capable of potentiating neuronal survival mechanisms. The ERK signaling pathway has a dual role in cell death and survival.

#### 3.3.4 Sensitivity analysis

For the acquisition of empirical importance of model parameters and entities upon each other, the whole TNF-*α* mediated sphingomyelin model was subjected to the sensitivity analysis as discussed earlier in the methodology (Section 2).

##### 3.3.4.1 Entities vs. parameters

**FIGURE 7:**
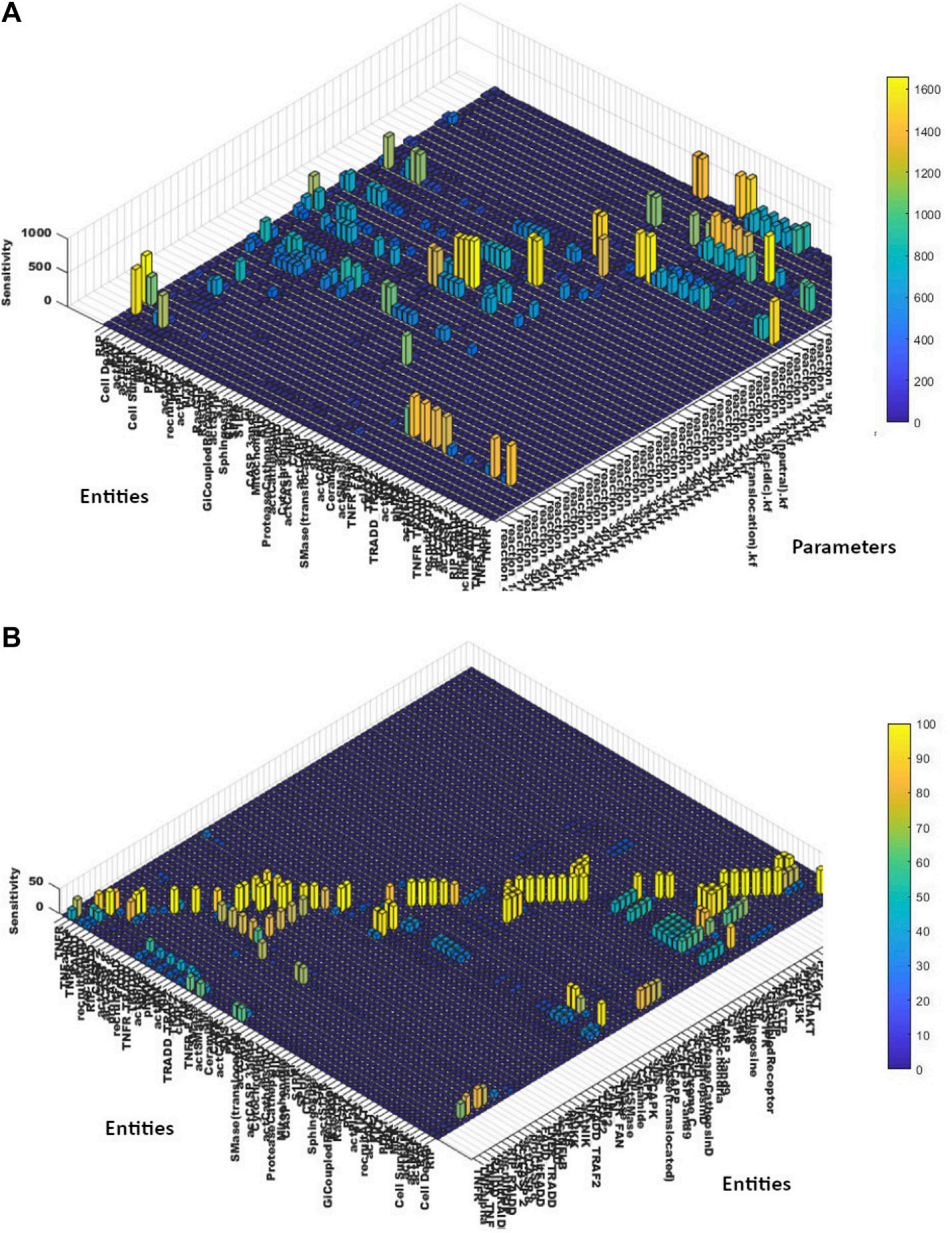
**(A)** Sensitivity Analysis of Entities vs. Parameters- The sensitivity analysis graph shows the sensitivities of the entities’ concentrations over the scale of 0–1800 in response to the interaction parameters of the TNF-*α* mediated sphingomyelin signaling pathway model. **(B)** Sensitivity Analysis of Entities vs. Entities- The sensitivity analysis graph shows the sensitivities of the entities’ concentrations over the scale of 0–100 towards other entities of the TNF-*α* mediated sphingomyelin signaling pathway model.

To study the effect of interaction parameters over entities concentrations, all the interaction parameters (kinetics) were taken as inputs, whereas all model entities were selected as outputs. The results of sensitivity analysis (Figure 7A) unveiled the importance of interaction 1 (Eq. (8) showing TNFR_TNF-*α* binding), interaction 15 (Eq. 14 showing TNFR_FAN formation) and interaction 27 (Eq. 17 showing Cathepsin D activation); as most of the model entities like SMase, PKC, BID, CASP 3 and 9 were found highly sensitive towards them having sensitivity values greater than 600. Moreover, high influence of interaction 27 (Eq. 17 showing Cathepsin D activation), interaction 49 (Eq. 18 showing RIP recruitment and interaction 51 (Eq. 19 showing ERK-mediated cell death) was observed on neuronal death. The sensitivity values of model entities against interactions parameters are given in the Supplementary Material Section 6. TNF-*α*, FAN, and SMase enzyme showed high sensitivity against interaction 1 (Eq. 8). FAN, SMase, TNF-*α*, PKC, and SMase_translocated were highly sensitive to interaction 15 (Eq. 14). Protease Cathepsin D, BID, CASP 3and9 were highly responsive to interaction 27 (Eq. (17)).

##### 3.3.4.2 Parameter scanning of the interactions having huge impact on neuronal apoptosis and survival

**FIGURE 8:**
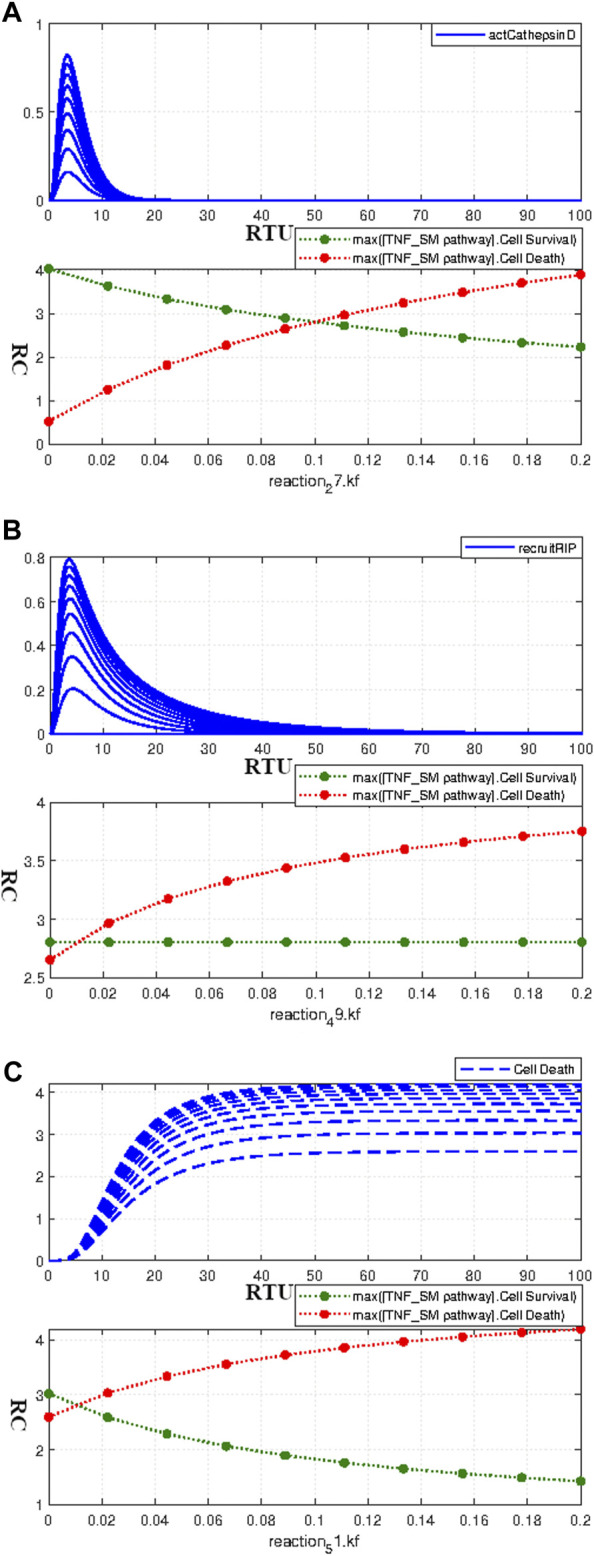
Paramater scanning of Interactions having huge impact on the Neuronal Survival and Apoptosis- **(A)** Paramater scanning of Interaction 27 (Eq. 17) representing Cathepsin D activation. **(B)** Paramater scanning for Interaction 49 (Eq. 18) representing RIP recruitment. **(C)** Paramater scanning for Interaction 51 (Eq. 19) representing ERK-mediated Cell Death. The increasing parameter value of the interactions representing Cathepsin D activation and ERK-mediated Cell Death over the range of 0–0.2 enhances neuronal apoptosis and decreases neuronal survival. However, the increasing parameter value of the interactions representing recruitment of RIP only enhances neuronal apoptosis without impacting neuronal survival.

Ceramide is responsible for the activation of protease cathepsin D, which is given by the interaction 27 in the model and Eq. 17. The neuronal apoptosis was found highly sensitive to this interaction as determined in the Section 3.3.4.1. The adjusted kinetic value for this interaction was taken as 0.1. The parameter scanning results in Figure 8A show the perfect conjunction of survival and apoptotic curves at the value 0.1. However, the change in parameter value profoundly affects the homeostatic state of the model and causes a profound increase in the relative difference with increasing or decreasing kinetic values.

$$ProteaseCathepsinD\;+\;Ceramide\;\;\overset{kf=0.1\left(adjusted\right)}{\to }\;\;actCathepsinD$$ (17)

$$RIP\;+\;TNFR_{T}RADD\;\;\overset{kf=0.01\left(adjusted\right)}{\to }\;\;recruitRIP$$ (18)

$$actERK\;+\;\;\;\overset{kf=0.01\left(adjusted\right)}{\to }\;\;\;\;CellDeath$$ (19)

The sensitivity analysis results in Section 3.3.4.1 determined the high influence of RIP recruitment on neuronal apoptosis. The recruitment of RIP *via* TRADD is given by interaction 49 (Eq. 18) with adjusted kinetics of 0.01. The parameter scanning results for this interaction, as given by Figure 8B, clearly show that an increase in parameter value causes an increase in neuronal apoptosis. Moreover, the higher kinetic values also inflate the relative difference between survival and apoptotic curves. ERK signaling cascade contributes both to neuronal survival as well as in apoptosis. The ERK-mediated neuronal apoptosis is indicated by interaction 51 (Eq. 19) with the adjusted kinetic value of 0.01. The parameter scanning results for this interaction as given by Figure 8C are following the sensitivity results as described in Section 3.3.4.1. The increasing parameter value increases apoptosis and decreases neuronal survival, thus, leading to a large relative difference among them with a rate increase. Figures 8A–C show parameter scanning for interactions 17, 18 and 19 respectively. The sensitivity analysis results as discussed in Section 3.3.4.1 revealed their high influence on neuronal death and survival. The parameter scanning curves for these interactions further conform to their significance as the change in their values causes drastic effects on neuronal survival and death.

##### 3.3.4.3 Entities vs. entities

Similarly, to study the influence of model entities concentrations over the other entities, the sensitivity values of model entities towards other entities are calculated (given in the Supplementary Material Section 7). The results (as evident from Figure 7B), revealed the significant sensitivity of neuronal death towards recruitRIP, RIP_RAIDD, actCASP8, actCASP2, actCASP3and9, cytochrome C, actBID and actCathepsinD. Moreover, neuronal survival was found more sensitive to actCAPK, RasGTP, actAKT, actERK, actMEK, actRAF. Apart from observing the sensitivities of neuronal death and survival against model parameters and entities, we also noticed a significant impact of model entities over the other entities. NF*κ*B, IKK, and NIK were found highly sensitive to TRADD_TRAF2 complex with the sensitivity values of 45.39, 46.84, and 48.33, respectively. Moreover, SMase enzyme and PKC showed high sensitivities against TNFR_FAN with the values of 49.04 and 47.4, respectively.

### 3.4 Dose estimation of drug and its response

#### 3.4.1 Effect of etanercept

**FIGURE 9:**
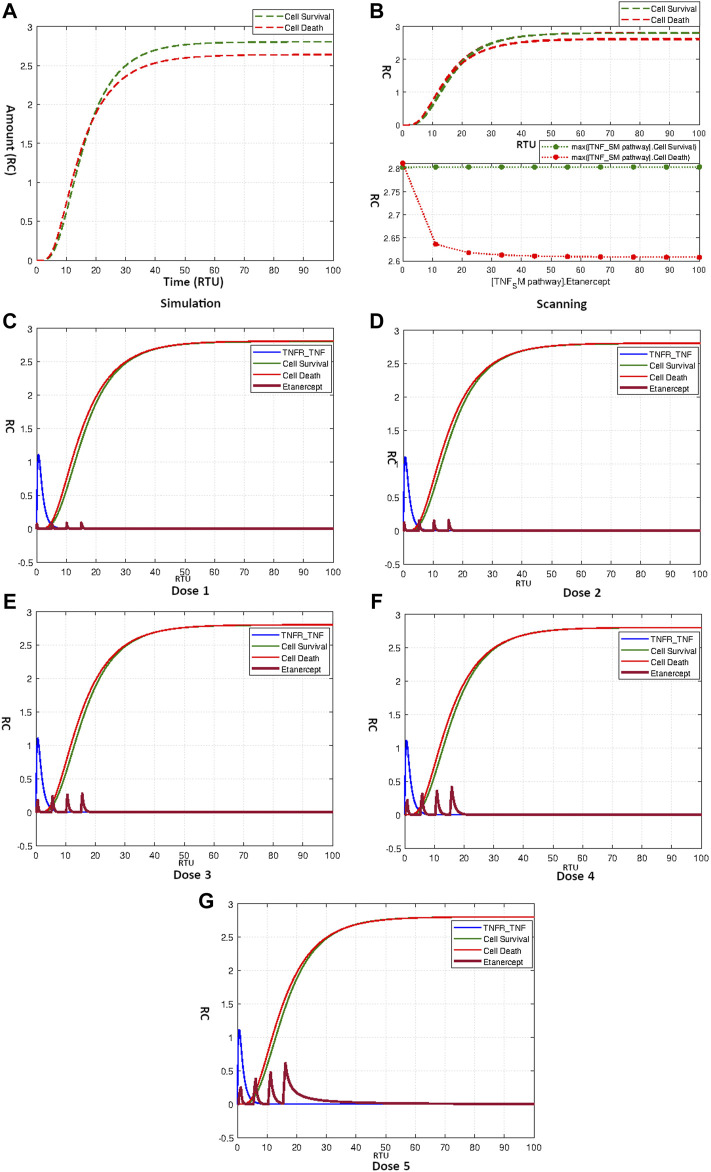
**(A)** Simulation showing effect of Etanercept on Neuronal Survival and Death. **(B)** Scanning of Etanercept performed over range 0–100 shows only minor decrease in cell death. **(C–G)** Figures c to g represent the dosage graphs for Etanercept according to Table 4. Dosages **(C–G)** have no obvious effect on neuronal death and survival.

The simulation result shows that Etanercept doesn’t produce a fierce effect on neuronal apoptosis for the current model (Figure 9A). The scanning result of Etanercept over a concentration range of 0–100 shown in Figure 9B further fortifies the fact of little efficacy of Etanercept for the current model. Moreover, neuronal survival remains completely unaffected by the application of Etanercept. Curves in Figures 9C–E show the repeated dosage effects of Etanercept with different start times and amounts as mentioned in Table 4. The outcomes clearly indicate the accumulation of Etanercept with increasing concentrations without prominently disturbing the balanced state of neurons.

**TABLE 4:** Application of Drugs using Repeat Dose.

Drugs	Doses	Start Time	Amount	Rate (k)	Interval	Repeat Count
Etanercept (10^–12^) (Chembl1201572)	Dose 1	0	0.1	1	5	3
	Dose 2	0.1	0.2	1	5	3
	Dose 3	0.2	0.4	1	5	3
	Dose 4	0.3	0.6	1	5	3
	Dose 5	0.4	0.8	1	5	3
Nivocasan (Chembl2105721)	Dose 1	0	0.1	1	5	3
	Dose 2	0.1	0.2	1	5	3
	Dose 3	0.2	0.4	1	5	3
	Dose 4	0.3	0.6	1	5	3
	Dose 5	0.4	0.8	1	5	3
Scyphostatin (10^–6^) (Chembl418376)	Dose 1	0	0.1	1	5	3
	Dose 2	0.1	0.2	1	5	3
	Dose 3	0.2	0.4	1	5	3
	Dose 4	0.3	0.6	1	5	3
	Dose 5	0.4	0.8	1	5	3

#### 3.4.2 Effect of nivocasan

**FIGURE 10:**
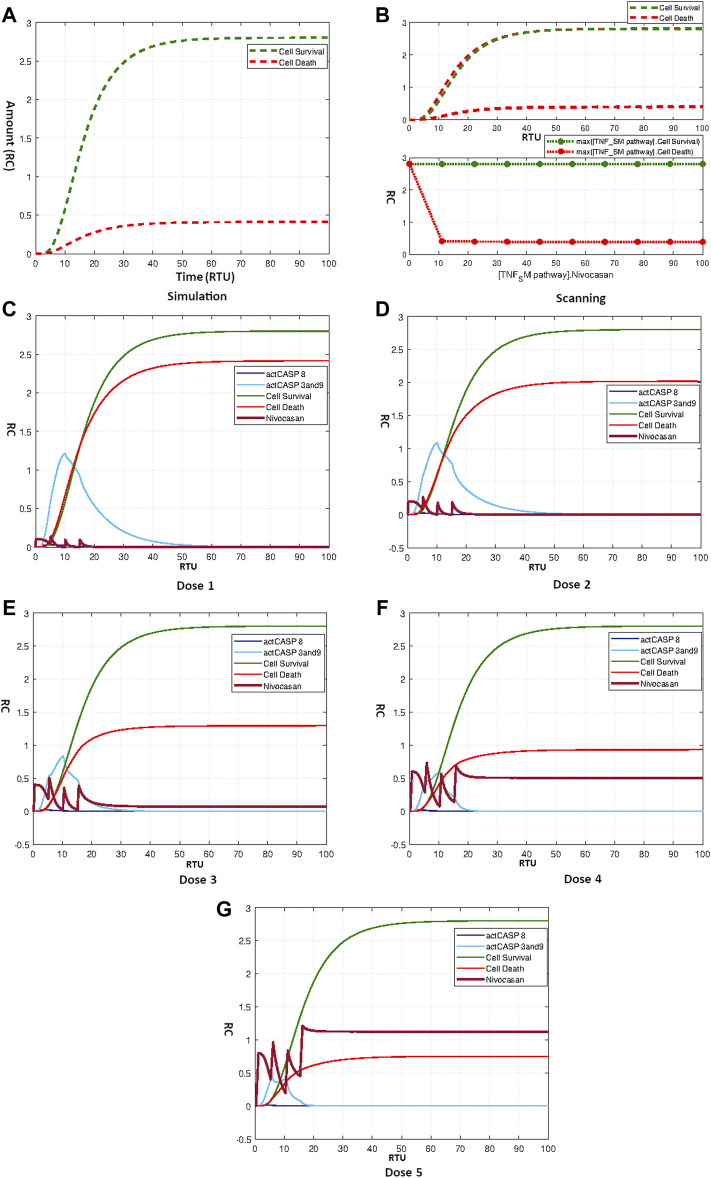
**(A)** Simulation showing effect of Nivocasan on Neuronal Survival and Death. **(B)** Scanning of Nivocasan. **(C–G)** Figures c to g represent the dosage graphs for the Nivocasan as according to Table 4. Dosages **(C–G)** show obvious effect on neuronal death.

As reported earlier in Section 3.3.4.3, the sensitivity analysis showed the high significance of caspase 8 and caspase 9 towards neuronal apoptosis. We repositioned the Nivocasan to the TNF-*α* mediated sphingomyelin signaling pathway model to analyze the model’s response towards caspase inhibitor. The simple simulation of model treated with Nivocasan with the initial amount 3 is given by Figure 10A. The caspase 8 and caspase 9 inhibition due to the application of Nivocasan results in lowering neuronal apoptosis with the difference of more than two folds.

Scanning was also performed for Nivocasan over the concentration range of 0–100. The corresponding results in Figure 10B show that increasing the amount of Nivocasan reduced the apoptotic mechanism up to a minimum point. The highest effect of Nivocasan is achieved for concentration 10 where apoptosis is minimum with the relative difference of about 2.5 folds. The application of repeated dosage of Nivocasan as according to the Table 4 produces graphs as given by Figures 10C–G. It is evident from the results that the effective dosage of Nivocasan can reduce neuronal apoptosis by creating a relative difference of about 2.5 folds between neuronal survival and neuronal death.

#### 3.4.3 Effect of scyphostatin

**FIGURE 11:**
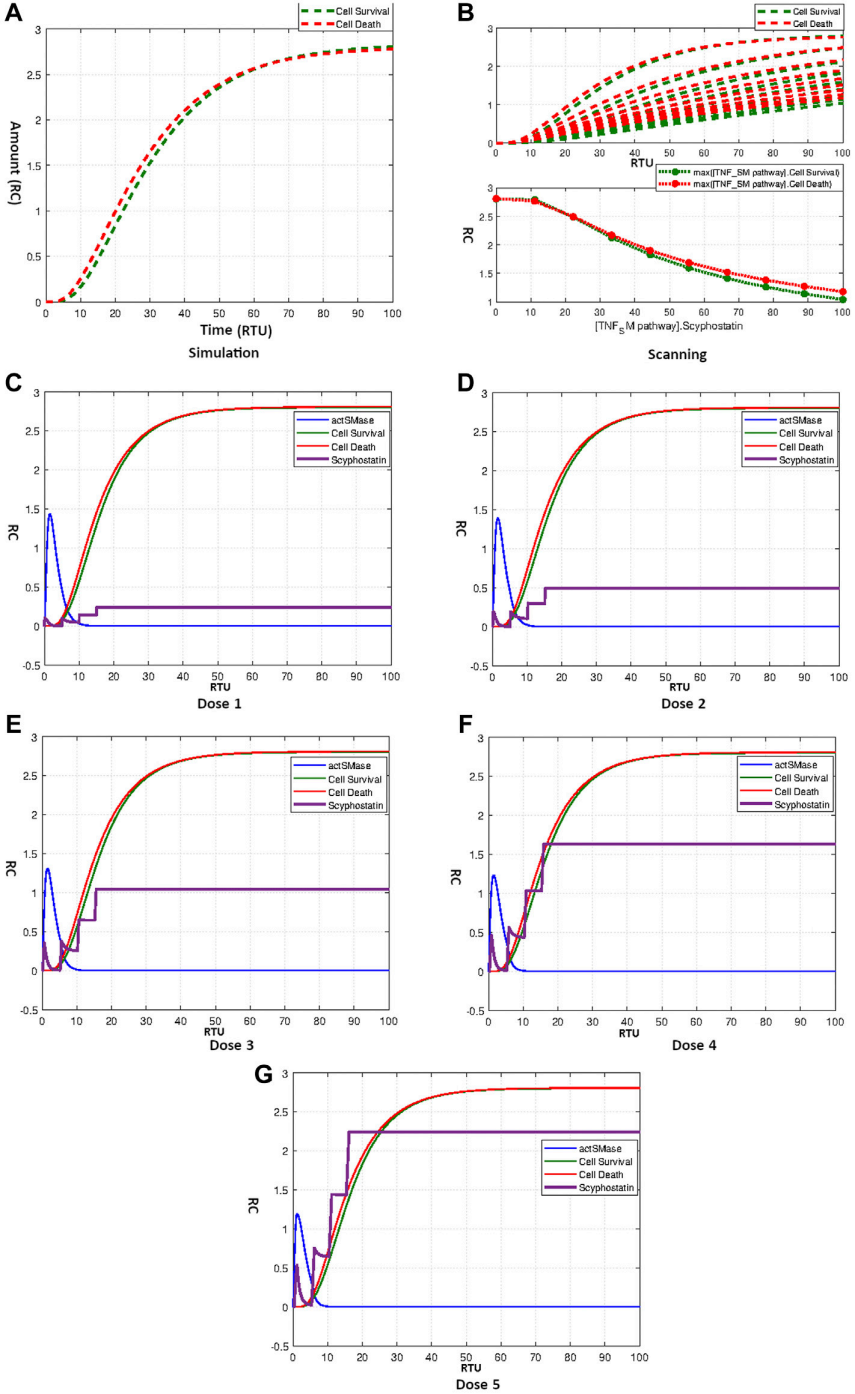
**(A)** Simulation showing effect of Scyphostatin on Neuronal Survival and Death. **(B)** Scanning of Scyphostatin. **(C–G)** Figures c to g represent the dosage graphs for Scyphostatin according to Table 4. Dosage **(C–G)** have no obvious effect on neuronal death and survival.

Figure 11A indicates that Scyphostatin fails to disturb the hypothesized equilibrium state of the model. The curves in Figure 11B show the scanning effect of Scyphostatin from concentration 0–100 for TNF-*α* mediated sphingomyelin signaling pathway model. It is evident from the results that increasing the concentration of drugs lowers both cell survival and cell death without affecting the overall homeostasis. The results of repeated dosages of Scyphostatin according to Table 4 four are given by Figures 11C–G. The results follow the speculations derived from Scyphostatin’s simulation and scanning. The model remains unaffected from repeat dosages with different starting times and amounts and retains its balanced state.

## 4 Discussion

The growing number of individuals affected by neurodegenerative disorders worldwide was a reason to focus and study diversified aspects of neurological diseases using a modeling approach. The severity of nearly all neuropathological diseases in AD, PD, and MS eventually leads to raised levels of TNF-*α* owing to enhanced glial cell production (Sharma et al., 2012; Sawada et al., 2006; Xu et al., 2015). The modeling of the sphingolipid metabolic pathway in association with TNF-*α* signaling provides a novel viewpoint on the behavior of various proteins, enzymes, and sphingolipids under homeostatic and pathological circumstances of neurons. Like sphingolipids, an up-regulated TNF-*α* expression has also been associated with the causation as well as the progression of several neurodegenerative diseases of the central nervous system (CNS) (Chitnis and Weiner, 2017). TNF-*α*, in alliance with its receptors, instigates the number of signaling pathways, including the sphingomyelin pathway (Olmos and Lladó, 2014). The literature studies in the subsequent part of discussion show the implication of cytokines and sphingolipid metabolites in numerous neuro-pathologies, making them robust targets for system-level studies.

In this study, the representational model of the TNF-*α* mediated sphingomyelin signaling system is modeled in Simbiology, which includes 73 entities and 51 interactions, to examine the linkage and interactions holistically. We built the model integrating data from the literature and KEGG, and the input concentrations of model entities were calculated using microarray data analysis. The kinetic rates of the interactions were set between the range 0.01–0.1 such that the system could depict the homeostatic balanced state of neuronal survival and apoptotic mechanisms. The values of kinetic parameters for model’s interactions were adjusted due to lack of kinetic values from literature. Therefore, relative time unit “RTU” was employed as a unit of time rather than the specific units. The simulation run of the model explicitly manifested the time and concentration of model entities during their recruitment or activation. Moreover, the simulation enabled us to track the minor details of even minute changes in entities concentrations over time. Figure 5 showed the hypothesized stable (balanced) state of TNF-*α* mediated sphingomyelin and related signaling pathways exhibited by neurons during normal circumstances.

The network analysis of the model using Cytoscape tool reveals the system’s essential components (adaptor proteins, enzymes, and chemicals) based on their betweenness and closeness centralities. The betweenness centralities of model entities were found in the following descending order: ceramide (0.078) < actSMase (0.051) < GiCoupled Receptor (0.031) < actS1PR (0.03) < sphingosine (0.0298) < recruitFADD (0.0292) < S1P(0.028) < TNFR_TRADD (0.023); evincing the high importance of ceramide among all other entities. It can be associated with the elevated levels of ceramide in the brain samples from patients affected by AD and other neurodegenerative disorders contributing to their pathophysiology (Filippov et al., 2012; Alessenko and Albi, 2020). The sphingolipids including ceramide, sphingomyelinase, and sphingosine with high betweeness centralities serve as prognostic biomarkers of neurodegeneration because of their increased levels in the AD brain (van Kruining et al., 2020). Moreover, the closeness centrality measure manifested the high closeness of actCASP2, actCASP8, cIAP2, actCAPP, actCASP 3and9, actAKT, actERK, cytochrome C, RIP_RAIDD, actNFkB, CAPP, recruitAKT, and PDK1 among all other entities of the model with closeness centrality values ranging from 1 to 0.63. Matsui et al. (2006) provided evidences of literature studies showing an increased activation of caspases 3, 8, and 9 in AD brain samples (Matsui et al., 2006).

The scanning results of elements with high betweenness centrality values further signified them being the most crucial components in the TNF-*α*-mediated sphingomyelin signaling pathway. The high impact of recruitFADD and TNFR_TRADD was observed on the neuronal apoptosis, whereas Gi-coupled receptor, actS1PR, Sphingosine, and S1P were found to produce a high influence on neuronal survival. Zhao et al. (2003) reported the elevated levels of both TNF-*α* and TRADD (being proapototic elements) in AD patients (Zhao et al., 2003). However, the current study inferred the dual role of ceramide, both on neuronal survival and apoptosis (Posse de Chaves, 2006), which suggests the multipotential function of ceramide both in neuronal survival and apoptosis (Toman et al., 2000). Neuronal apoptosis was more affected by the recruitFADD with a betweenness value of 0.0292 when compared to TNFR_TRADD (betweenness value 0.023). It suggests the increased levels of FADD protein that occur in the midbrain of PD patients. (Gorman, 2008). Moreover, Ferguson et al. (2020) found the high levels of FADD in the post-mortem brain tissue from the BA21 region of African Americans affected by AD (Ferguson et al., 2020). Also, Wu et al. (2005) presented the first demonstration of enhanced FADD expression in the AD brain (Wu et al., 2005). It can also be observed that among the Gi-coupled receptor, actS1PR, sphingosine, and S1P, the increased activation of the Gi-coupled receptor highly influenced neuronal survival. The increasing concentrations of actCASP2, actCASP8, actCASP 3and9, cytochrome C, and RIP_RAIDD with the high closeness centrality values appeared as entities chiefly affecting the neuronal apoptosis. The scanning of actCASP2, actCASP8, and actCASP3and9 showed their profound influence on neuronal apoptosis due to the high centrality value.

The parameter scanning allowed comparison of kinetic parameters used in the current study with some of kinetic values according to the literature (Cho et al., 2003). The variations among these kinetics were marked bearable as they lied within tolerable ranges of variations and showed no drastic effects on the model. Scanning was done for the kinetic parameter values for interactions 1 (Eq. 8), 2 (Eq. 9), 7 (Eq. 11), 8 (Eq. 10), 9 (Eq. 12), 12 (Eq. 13), 15 (Eq. 14), 31 (Eq. 15) and 38 (Eq. 16) and compared with some known parameter kinetics to determine their effect on the homeostatic state of the model. Parameter scanning over the range 0–0.2 of the mentioned interactions showed that kinetic rate parameters governing TNF_TNFR formation, recruitment of RAIDD, TNFR-TRADD formation, recruitment of FADD, TRADD_TRAF2 formation, TNFR_FAN association, S1PR activation, and recruitment of AKT only cause minor alterations in the balanced state of the system over the given range. The exceprional behavior of NF*κ*B can be attributed to its contribution in promoting cell survival *via* its inhibitory action on caspase 8.

Sensitivity analysis allowed us to determine the effects of all the interaction parameters (kinetics) on the model entities, and also among the entities themselves. Sensitivity analysis of entities versus parameters indicated the high impact of interaction 1 representing TNF-*α* binding with TNFR and interaction 15 (Eq. 14) representing TNFR_FAN association over SMase enzyme with the sensitivity values of 613.7 and 657.6 (over the scale ranging from 0 to 700), respectively. The Jurkat T lymphocytes showed a significant increase in the activity of acidic SMase and a minor increase in the activity of neutral SMase (NSMase) when exposed to TNF-*α* (for 5 min and 7 h, respectively) due to enhanced TNF-*α*/TNFR1 binding (Church et al., 2005). Moreover, the FAN constitutively binds with neutral sphingomyelinase activation domain (NSD) of TNFR1 and triggers activation of NSMase (Montfort et al., 2010). We also witnessed the high impact of interaction 15 (Eq. 14) on PKC with a sensitivity value of 636.3. BID and CASP 3and9 were found highly sensitive to interaction 27 (Eq. 17) (representing ceramide triggered activation of cathepsin D) with values 682 and 641.6, respectively. This infers the role of ceramide induced activation of cathepsin D to form truncated BID (tBID, referred as actBID in model) from BID Poulaki and Giannouli (2022). Neuronal survival was found sensitive to interaction 51 (Eq. 19), interaction 40, interaction 18, interaction 43 and interaction 34 of the model (shown in the Figure 7A and Supplementary Material Section 6) with the sensitivity values 483.3, 302.1, 295.7, 234.7, and 209.6, respectively. These interactions represent ERK-mediated neuronal apoptosis, the CAPP-mediated inhibition of AKT, CAPK activation, ERK-mediated neuronal survival, and conversion of RasGTP to RasGDP, respectively. Finegan et al. (2009) provided the genetic evidence for the involvement of ERK5 in regulating neuronal survival (Finegan et al., 2009). However, neuronal death was most affected by interaction 51 (Eq. 19), interaction 27 (17), interaction 49 (18) and interaction 28 representing ERK-mediated neuronal apoptosis, ceramide triggered activation of cathepsin D, recruitment of RIP *via* TNFR_TRADD and caspase 3,9 activated cell death with sensitivity values 669.7, 471.4, 418.9 and 220.5 respectively. Sensitivity analysis of parameters versus entities showed highly influence of interactions governing cathepsin D activation (Eq. 17), recruitment of RIP (Eq. 18) and ERK-mediated neuronal death (Eq. 19) on neuronal apoptosis. In the literature, we have evidence of the dual role of ERK signaling pathway in neuronal survival and apoptosis (Li et al., 2014). The parameter scanning results of interactions 27 (Eq. 17), 49 (Eq. 18) and 51 (Eq. 19) were perfectly in accordance with their sensitivity effect on apoptosis and survival.

Finally, the efficacy of different FDA-approved drugs was determined by analyzing the response of the sphingomyelin signaling pathway model towards different drugs’ dosages. The appropriate drugs were selected based on their applicability and effectiveness for the relevant targets in the current model. Etanercept, Nivocasan, and Scyphostatin were among the drugs whose dosages were examined. Each drug’s results and effects were tracked by scanning and repeated dosing. It was found that Etanercept and Scyphostatin had no effect on the model’s equilibrium, and led to their accumulation. However, Nivocasan’s irreversible inhibitory actions on caspase 8 and caspase 9 significantly reduced neuronal apoptosis to a large degree (Xie et al., 2020). Caspase 8 is an important mediator of neuronal apoptosis during neurodegenerative diseases (Zhang et al., 2020), whereas Blandini et al., (2006) reported enhanced activity of caspase 9 in PD patients (Blandini et al., 2006). It is worth mentioning that caspases 8 and 9 showed high closeness centrality *via* network analysis, manifesting their importance in TNF-*α* mediated sphingomyelin signaling pathway. Avrutsky and Troy (2021) examined the role of caspase 9 as a multimodal therapeutic target for a range of diseases including AD (Avrutsky and Troy, 2021). Hence, the significance of Nivocasan was pronounced due to its applicability on caspase 8 and caspase 9.

As mentioned earlier, different neuropathologies including immune disease, trauma, and inflammation may lead to rapid and irreversible neurological damage. Different pharmacological treatments have been formulated and applied for the aforementioned neurological disorders. Etanercept, a TNF antagonist is used as a TNF blocker in order to minimize the neurological damage mediated by TNF-dependent processes (Kondziella and Waldemar, 2017). Figure 9 and Figure 11 show the response of the model towards the drugs Etanercept and Scyphostatin. The simulation result shows that Etanercept doesn’t produce any apparent reduction in neuronal apoptosis for the current model. Likewise, the drug Scyphostatin fails to show any prominent effect on neuronal apoptosis or survival. On the other hand, a precise concentration of Nivocasan produces a magnificent curtailment in neuronal apoptosis.

## 5 Conclusion and future perspectives

Neurological disorders are the leading cause of disabilities and deaths worldwide. Most of them are neurodegenerative and are responsible for initiating neuroinflammatory responses. The prevailing conditions of neurological injuries induce sustained neuroinflammation, which ultimately leads to neuronal loss and death. The formulation of common therapeutic strategies for all neurodegenerative disorders at the later inflammatory stages can prevent the most damaging state of neuronal loss. The integration of common neuroinflammatory responses with the sphingolipid signaling pathway, and their quantitative analysis *via* computational modeling approach, provided a broad spectrum of devising therapeutic targets. The findings identified the number of caspases (CASP2, CASP8, and CASP9) and sphingolipids (S1P) as potent proteins having therapeutic significance at subsequent stages of the disease. Moreover, the drug dosage analysis evaluated the efficacy of three drugs, i.e., Etanercept, Nivocasan, and Scyphostatin, at the level of neuroinflammatory responses.

The application of Etanercept and Scyphostatin with varying dosages failed to suppress neuronal apoptosis, or in other words, did not disturb the homeostatic balanced state of neurons. These results manifested that repurposing of the two drugs was impracticable at later stages of neuroinflammation. However, the drug dosage analysis indicated Nivocasan’s exertion of the inhibitory mechanism at caspase 8 and caspase 9, which significantly altered the system homeostasis. It shows that the neuronal homeostatic state is prone to transmute at the later stages of inflammation when encountered with caspase 8 and caspase 9 inhibitors. The current study (model generated and analyzed using AD data) can be extended to other neurodegenerative diseases like PD and MS to analyze the effects of various entities and interaction parameters and formulate effective therapeutics at their furthest levels.

## Data Availability

Publicly available datasets were analyzed in this study. This data can be found here: https://www.ncbi.nlm.nih.gov/geo/query/acc.cgi?acc=GSE36980.

## Nomenclature

actAKT: Protein kinase B (activated)
actBID: BH3 Interacting-domain Death agonist (activated)
actCAPK: Ceramide Activated Protein Kinase (activated)
actCAPP: Ceramide Activated Protein Phosphatase (activated)
actCASP2: Caspase 2 (activated)
actCASP3and9: Caspases 3 and 9 (activated)
actCASP8: Caspase 8 (activated)
actCathepsinD: Cathepsin D (activated)
actMEK: Mitogen-activated Protein Kinase (activated)
actNF*κ*B: Nuclear Factor kappa B (activated)
actNIK: NF-*κ*B-Inducing Kinase (activated)
actPI3K: phosphoinositide 3-kinase (activated)
actRAF: Rapidly Accelerated Fibrosarcoma kinase (activated)
actS1PR: Sphingosine-1-Phosphate Receptor (activated)
actSMase: Sphingomyelinase (activated)
AD: Alzheimer’s Disease
AKT: Protein Kinase B
ASD: Autism Spectrum Disorder
BID: BH3 Interacting-Domain Death Agonist
CAPK: Ceramide Activated Protein Kinase
CAPP: Ceramide Activated Protein Phosphatase
CASP 2: Caspase 2
CASP 3 and 9: Caspase 3 and 9
CDase: Ceramidase
cIAP2: Inhibitor of Apoptosis Protein
DD: Death Domain
ERK: Extracellular Signal-Regulated Kinase
FADD: Fas-Associated Protein with Death Domain
FAN: Factor Associated with Neutral Sphingomyelinase Activation
GAP: GTPase-Activating Protein
GDP: Guanosine Diphosphate
GTP: Guanosine Triphosphate
Gi: G-protein Alpha Subunit
Gi Coupled Receptor: G-protein Coupled Receptor
IKK: Inhibitor of Nuclear Factor kappa-B Kinase
MEK: Mitogen-activated Protein Kinase
MS: Multiple sclerosis
NF*κ*B: Nuclear Factor Kappa B
NIK: NF-*κ*B Inducing Kinase
PD: Parkinson’s Disease
PDK1: Phosphoinositide-Dependent Kinase-1
PhIKK: Inhibitor of Nuclear Factor Kappa-B Kinase (phosphorylated)
PI3K: phosphoinositide 3-kinase
PIP2: Phosphatidylinositol 4,5-Bisphosphate
PIP3: Phosphatidylinositol (3,4,5)-Trisphosphate
PKC: Protein kinase C
proCASP 8: Procaspase-8
ProtCathepD: Protease Cathepsin D
RAF: Rapidly Accelerated Fibrosarcoma
RAIDD: RIP-Associated Protein with a Death Domain
RasGDP: GDP-bound state of Ras
RasGTP: GTP-bound state of Ras
recruitRAIDD: recruited RIP-Associated Protein with a Death Domain
recruit AKT: recruited Protein Kinase B
recruit FADD: recruited Fas-Associated Protein with Death Domain
recruit RIP: recruited Receptor-Interacting Protein Kinase
RIP: Receptor-Interacting Protein Kinase
RIP_RAIDD: RIP RAIDD complex
S1P: Sphingosine 1 Phosphate
S1PR: Sphingosine-1-Phosphate Receptor
SM: Sphingomyelin
SMase: Sphingomyelinase
SMase(translocated): Sphingomyelinase (translocated)
SMS: Sphingomyelin Synthase
SPHK: Sphingosine Kinase
TNF-*α*: Tumor Necrosis Factor-*α*
TNFR: Tumor Necrosis Factor Receptor
TNFR_FAN: Complex of TNFR and FAN
TNFR_TNF: Complex of TNFR and TNF
TNFR_TRADD: Complex of TNFR and TRADD
TRADD: Tumor Necrosis Factor Receptor type 1-Associated Death Domain Protein
TRADD_TRAF2: Complex of TRADD
TRAF2_TRAF2: TNF Receptor-Associated Factor 2
RTU: Relative Time Unit
RC: Relative Concentration
