# Supplementary material for: System level modeling and analysis of TNF-α mediated sphingolipid signaling pathway in neurological disorders for the prediction of therapeutic targets
Source: Front Physiol. 2022 Aug 19;13:872421. doi: 10.3389/fphys.2022.872421 (PMC9437628; doi:10.3389/fphys.2022.872421)
Supplement: Supplementary file 1 [file DataSheet2.PDF]

1 All the Interactions of TNF-α-mediated sphingomyelin signaling pathway model

S.No	Interactions	Reaction Rate
1	TNFR + TNFα $\longrightarrow$ TNFR_TNF	kf*TNFR*TNFα
2	TNFR_TNF + RAIDD $\longrightarrow$ recruitRAIDD	kf*TNFR_TNF*RAIDD
3	recruitRAIDD + recruitRIP $\longrightarrow$ RIP_RAIDD	kf*recruitRAIDD*recruitRIP
4	RIP_RAIDD + [CASP 2] $\longrightarrow$ [actCASP 2]	kf*RIP_RAIDD*[CASP 2]
5	[actCASP 8] $\longrightarrow$ [Cell Death]	kf*[actCASP 8]
6	recruitFADD + [proCASP 8] $\longrightarrow$ [actCASP 8]	kf*recruitFADD*[proCASP 8]
7	TNFR_TRADD + FADD $\longrightarrow$ recruitFADD	kf*TNFR_TRADD*FADD
8	TRADD + TNFR_TNF $\longrightarrow$ TNFR_TRADD	kf*TRADD*TNFR_TNF
9	phIKK + NFkB $\longrightarrow$ actNFkB	kf*phIKK*NFkB
10	actNIK + IKK $\longrightarrow$ phIKK	kf*actNIK*IKK
11	TRADD_TRAF2 + NIK $\longrightarrow$ actNIK	kf*TRADD_TRAF2*NIK
12	TRAF2 + TNFR_TRADD $\longrightarrow$ TRADD_TRAF2	kf*TRAF2*TNFR_TRADD
13	actNFkB $\longrightarrow$ cIAP2	kf*actNFkB
14	FAN + TNFR $\longrightarrow$ TNFR_FAN	kf*FAN*TNFR
15	TNFR_FAN + SMase $\longrightarrow$ actSMase	kf*TNFR_FAN*SMase
16	Ceramide + CAPK $\longrightarrow$ actCAPK	kf*Ceramide*CAPK
17	recruitFADD + SMase $\longrightarrow$ actSMase	kf*recruitFADD*SMase
18	Ceramide + SMS $\longrightarrow$ SM + SMS	kf*Ceramide*SMS
19	actSMase + PKC $\longrightarrow$ [SMase(translocated)]	kf*actSMase*PKC
20	SM + [SMase(translocated)] $\longrightarrow$ Ceramide + [SMase(translocated)]	kf*SM*[SMase(translocated)]
21	CAPP + Ceramide $\longrightarrow$ actCAPP	kf*CAPP*Ceramide
22	[Cytochrome C] + [CASP 3and9] $\longrightarrow$ [actCASP 3and9]	kf*[Cytochrome C]*[CASP 3and9]
23	actBID + Mitochondria $\longrightarrow$ [Cytochrome C]	kf*actBID*Mitochondria
24	actCathepsinD + BID $\longrightarrow$ actBID	kf*actCathepsinD*BID
25	ProteaseCathepsinD + Ceramide $\longrightarrow$ actCathepsinD	kf*ProteaseCathepsinD*Ceramide
26	[actCASP 3and9] $\longrightarrow$ [Cell Death]	kf*[actCASP 3and9]
27	CDase + Ceramide $\longrightarrow$ Sphingosine + CDase	kf*CDase*Ceramide
28	Sphingosine + SPHK $\longrightarrow$ S1P + SPHK	kf*Sphingosine*SPHK
29	S1P + S1PR $\longrightarrow$ actS1PR	kf*S1P*S1PR
30	actS1PR + Gi $\longrightarrow$ GiCoupledReceptor	kf*actS1PR*Gi
31	RasGDP + GiCoupledReceptor $\longrightarrow$ RasGTP	kf*RasGDP*GiCoupledReceptor
32	RasGTP + GAP $\longrightarrow$ RasGDP	kf*RasGTP*GAP
33	Sphingosine + CDase $\longrightarrow$ Ceramide + CDase	kf*Sphingosine*CDase
34	PI3K + GiCoupledReceptor $\longrightarrow$ actPI3K	kf*PI3K*GiCoupledReceptor
35	actPI3K + PIP2 $\longrightarrow$ PIP3	kf*actPI3K*PIP2
36	PIP3 + AKT $\longrightarrow$ recruitAKT	kf*PIP3*AKT
37	recruitAKT + PDK1 $\longrightarrow$ actAKT	kf*recruitAKT*PDK1
38	actCAPP + recruitAKT + PDK1 $\longrightarrow$ null	kf*actCAPP*recruitAKT*PDK1

39	actAKT + actCathepsinD + BID $\longrightarrow$ null	kf*actAKT*actCathepsinD*BID
40	actAKT + [Cytochrome C] + [CASP 3and9] $\longrightarrow$ null	kf*actAKT*[Cytochrome C]*[CASP 3and9]
41	actERK $\longrightarrow$ [Cell Survival]	kf*actERK
42	actMEK + ERK $\longrightarrow$ actERK	kf*actMEK*ERK
43	actRAF + MEK $\longrightarrow$ actMEK	kf*actRAF*MEK
44	RAF + RasGTP $\longrightarrow$ actRAF	kf*RAF*RasGTP
45	[actCASP 2] $\longrightarrow$ [Cell Death]	kf*[actCASP 2]
46	RIP + TNFR_TRADD $\longrightarrow$ recruitRIP	kf*RIP*TNFR_TRADD
47	actAKT $\longrightarrow$ [Cell Survival]	kf*actAKT
48	actERK $\longrightarrow$ [Cell Death]	kf*actERK
49	SM + actSMase $\longrightarrow$ Ceramide + actSMase	kf*SM*actSMase
50	actCAPK + RAF $\longrightarrow$ actRAF	kf*actCAPK*RAF
51	[actCASP 8] + cIAP2 $\longrightarrow$ null	kf*[actCASP 8]*cIAP2

2 Ordinary Differential Equations representing Simulations of Interactions of TNF-α-mediated sphingomyelin signaling pathway model

- d(TNFR)/dt = 1/[TNF_SM pathway]*(-ReactionFlux1 - ReactionFlux14)
- d(TNFalpha)/dt = 1/[TNF_SM pathway]*(-ReactionFlux1)
- d(TNFR_TNF)/dt = 1/[TNF_SM pathway]*(ReactionFlux1 - ReactionFlux2 - ReactionFlux8)
- d(RAIDD)/dt = 1/[TNF_SM pathway]*(-ReactionFlux2)
- d(recruitRAIDD)/dt = 1/[TNF_SM pathway]*(ReactionFlux2 - ReactionFlux3)
- d(recruitRIP)/dt = 1/[TNF_SM pathway]*(-ReactionFlux3 + ReactionFlux46)
- d(RIP_RAIDD)/dt = 1/[TNF_SM pathway]*(ReactionFlux3 - ReactionFlux4)
- d([CASP 2])/dt = 1/[TNF_SM pathway]*(-ReactionFlux4)
- d([actCASP 2])/dt = 1/[TNF_SM pathway]*(ReactionFlux4 - ReactionFlux45)
- d([actCASP 8])/dt = 1/[TNF_SM pathway]*(-ReactionFlux5 + ReactionFlux6 - ReactionFlux51)
- d([proCASP 8])/dt = 1/[TNF_SM pathway]*(-ReactionFlux6)
- d(recruitFADD)/dt = 1/[TNF_SM pathway]*(-ReactionFlux6 + ReactionFlux7 - ReactionFlux17)
- d(FADD)/dt = 1/[TNF_SM pathway]*(-ReactionFlux7)
- d(TNFR_TRADD)/dt = 1/[TNF_SM pathway]*(-ReactionFlux7 + ReactionFlux8 - ReactionFlux12 - ReactionFlux46)
- d(TRADD)/dt = 1/[TNF_SM pathway]*(-ReactionFlux8)
- d(actNFkB)/dt = 1/[TNF_SM pathway]*(ReactionFlux9 - ReactionFlux13)
- d(NFkB)/dt = 1/[TNF_SM pathway]*(-ReactionFlux9)
- d(phIKK)/dt = 1/[TNF_SM pathway]*(-ReactionFlux9 + ReactionFlux10)
- d(IKK)/dt = 1/[TNF_SM pathway]*(-ReactionFlux10)
- d(actNIK)/dt = 1/[TNF_SM pathway]*(-ReactionFlux10 + ReactionFlux11)

- d(NIK)/dt = 1/[TNF_SM pathway]*(-ReactionFlux11)
- d(TRADD_TRAF2)/dt = 1/[TNF_SM pathway]*(-ReactionFlux11 + ReactionFlux12)
- d(TRAF2)/dt = 1/[TNF_SM pathway]*(-ReactionFlux12)
- d(cIAP2)/dt = 1/[TNF_SM pathway]*(ReactionFlux13 - ReactionFlux51)
- d(FAN)/dt = 1/[TNF_SM pathway]*(-ReactionFlux14)
- d(TNFR_FAN)/dt = 1/[TNF_SM pathway]*(ReactionFlux14 - ReactionFlux15)
- d(SMase)/dt = 1/[TNF_SM pathway]*(-ReactionFlux15 - ReactionFlux17)
- d(actSMase)/dt = 1/[TNF_SM pathway]*(ReactionFlux15 + ReactionFlux17 - ReactionFlux19)
- d(SM)/dt = 1/[TNF_SM pathway]*(ReactionFlux18 - ReactionFlux20 - ReactionFlux49)
- d(Ceramide)/dt = 1/[TNF_SM pathway]*(-ReactionFlux16 - ReactionFlux18 + ReactionFlux20 - ReactionFlux21 - ReactionFlux25 - ReactionFlux27 + ReactionFlux33 + ReactionFlux49)
- d(CAPK)/dt = 1/[TNF_SM pathway]*(-ReactionFlux16)
- d(actCAPK)/dt = 1/[TNF_SM pathway]*(ReactionFlux16 - ReactionFlux50)
- d(PKC)/dt = 1/[TNF_SM pathway]*(-ReactionFlux19)
- d([SMase(translocated)])/dt = 1/[TNF_SM pathway]*(ReactionFlux19)
- d(actCAPP)/dt = 1/[TNF_SM pathway]*(ReactionFlux21 - ReactionFlux38)
- d(CAPP)/dt = 1/[TNF_SM pathway]*(-ReactionFlux21)
- d([actCASP 3and9])/dt = 1/[TNF_SM pathway]*(ReactionFlux22 - ReactionFlux26)
- d([Cytochrome C])/dt = 1/[TNF_SM pathway]*(-ReactionFlux22 + ReactionFlux23 - ReactionFlux40)
- d(actBID)/dt = 1/[TNF_SM pathway]*(-ReactionFlux23 + ReactionFlux24)
- d(actCathepsinD)/dt = 1/[TNF_SM pathway]*(-ReactionFlux24 + ReactionFlux25 - ReactionFlux39)
- d(ProteaseCathepsinD)/dt = 1/[TNF_SM pathway]*(-ReactionFlux25)
- d(BID)/dt = 1/[TNF_SM pathway]*(-ReactionFlux24 - ReactionFlux39)

- d(Mitochondria)/dt = 1/[TNF_SM pathway]*(-ReactionFlux23)
- d([CASP 3and9])/dt = 1/[TNF_SM pathway]*(-ReactionFlux22 - ReactionFlux40)
- d(Gi)/dt = 1/[TNF_SM pathway]*(-ReactionFlux30)
- d(S1PR)/dt = 1/[TNF_SM pathway]*(-ReactionFlux29)
- d(Sphingosine)/dt = 1/[TNF_SM pathway]*(ReactionFlux27 - ReactionFlux28 - ReactionFlux33)
- d(S1P)/dt = 1/[TNF_SM pathway]*(ReactionFlux28 - ReactionFlux29)
- d(actS1PR)/dt = 1/[TNF_SM pathway]*(ReactionFlux29 - ReactionFlux30)
- d(GiCoupledReceptor)/dt = 1/[TNF_SM pathway]*(ReactionFlux30 - ReactionFlux31 - ReactionFlux34)
- d(RasGDP)/dt = 1/[TNF_SM pathway]*(-ReactionFlux31 + ReactionFlux32)
- d(RasGTP)/dt = 1/[TNF_SM pathway]*(ReactionFlux31 - ReactionFlux32 - ReactionFlux44)
- d(GAP)/dt = 1/[TNF_SM pathway]*(-ReactionFlux32)
- d(PI3K)/dt = 1/[TNF_SM pathway]*(-ReactionFlux34)
- d(actPI3K)/dt = 1/[TNF_SM pathway]*(ReactionFlux34 - ReactionFlux35)
- d(PIP3)/dt = 1/[TNF_SM pathway]*(ReactionFlux35 - ReactionFlux36)
- d(recruitAKT)/dt = 1/[TNF_SM pathway]*(ReactionFlux36 - ReactionFlux37 - ReactionFlux38)
- d(actAKT)/dt = 1/[TNF_SM pathway]*(ReactionFlux37 - ReactionFlux39 - ReactionFlux40 - ReactionFlux47)
- d(PIP2)/dt = 1/[TNF_SM pathway]*(-ReactionFlux35)
- d(AKT)/dt = 1/[TNF_SM pathway]*(-ReactionFlux36)
- d(PDK1)/dt = 1/[TNF_SM pathway]*(-ReactionFlux37 - ReactionFlux38)
- d(ERK)/dt = 1/[TNF_SM pathway]*(-ReactionFlux42)
- d(MEK)/dt = 1/[TNF_SM pathway]*(-ReactionFlux43)
- d([Cell Survival])/dt = 1/[TNF_SM pathway]*(ReactionFlux41 + ReactionFlux47)
- d(actERK)/dt = 1/[TNF_SM pathway]*(-ReactionFlux41 + ReactionFlux42 - ReactionFlux48)

- d(actMEK)/dt = 1/[TNF_SM pathway]*(-ReactionFlux42 + ReactionFlux43)
- d(actRAF)/dt = 1/[TNF_SM pathway]*(-ReactionFlux43 + ReactionFlux44 + ReactionFlux50)
- d(RAF)/dt = 1/[TNF_SM pathway]*(-ReactionFlux44 - ReactionFlux50)
- d([Cell Death])/dt = 1/[TNF_SM pathway]*(ReactionFlux5 + ReactionFlux26 + ReactionFlux45 + ReactionFlux48)
- d(RIP)/dt = 1/[TNF_SM pathway]*(-ReactionFlux46)

3 Mass Action Kinetic Values used as Kinetic Parameters for Interactions of TNF-α-mediated sphingomyelin signaling pathway model

Interaction Rate Kinetic	Kinetic Value	Interaction Rate Kinetic	Kinetic Value
1.kf	0.1	29.kf	0.1
2.kf	0.1	30.kf	0.1
3.kf	0.1	31.kf	0.1
4.kf	0.1	32.kf	0.1
5.kf	0.1	33.kf	0.1
6.kf	0.1	34.kf	0.01
7.kf	0.01	35.kf	0.001
8.kf	0.1	36.kf	0.1
9.kf	0.1	37.kf	0.1
10.kf	0.1	38.kf	0.1
11.kf	0.1	39.kf	0.1
12.kf	0.1	40.kf	0.01
13.kf	0.1	41.kf	0.01
15.kf	0.1	42.kf	0.01
16.kf	0.1	43.kf	0.1
18.kf	0.1	44.kf	0.1
19.kf	0.1	45.kf	0.1
20.kf	0.01	46.kf	0.1
21.kf	0.1	47.kf	0.1
22.kf	0.1	49.kf	0.01
23.kf	0.1	50.kf	0.1
24.kf	0.1	51.kf	0.01
25.kf	0.1	17.kf	0.1
26.kf	0.1	14.kf	0.1
27.kf	0.1	48.kf	0.1
28.kf	0.1		

4 Values of Betweenness Centrality of the Entities of TNF-α-mediated sphingomyelin signaling pathway model

S.No.	Name	Betweenness Centrality	S.No.	Name	Betweenness Centrality
1	Ceramide	0.07859649	40	RasGDP	0.00105263
2	actSMase	0.05140351	41	TNFR	0
3	GiCoupledReceptor	0.03122807	42	TNFalpha	0
4	actS1PR	0.03	43	RAIDD	0
5	Sphingosine	0.02982456	44	CASP2	0
6	recruitFADD	0.02921053	45	CellDeath	0
7	S1P	0.02807018	46	proCASP8	0
8	TNFR_TRADD	0.02289474	47	FADD	0
9	actRAF	0.01833333	48	TRADD	0
10	actCathepsinD	0.01780702	49	NFkB	0
11	actPI3K	0.01754386	50	IKK	0
12	PIP3	0.01526316	51	NIK	0
13	actCAPK	0.01447368	52	TRAF2	0
14	actMEK	0.01412281	53	FAN	0
15	actBID	0.01254386	54	SMase	0
16	recruitAKT	0.0122807	55	CAPK	0
17	TNFR_FAN	0.01070175	56	SMS	0
18	RasGTP	0.01070175	57	PKC	0
19	TNFR_TNF	0.01017544	58	CAPP	0
20	CytochromeC	0.00991228	59	CASP3and9	0
21	actERK	0.00921053	60	Mitochondria	0
22	SM	0.00649123	61	BID	0
23	actAKT	0.00631579	62	ProteaseCathepsinD	0
24	SMase_translocated	0.00526316	63	CDase	0
25	actNIK	0.00473684	64	SPHK	0
26	phIKK	0.00438596	65	S1PR	0
27	TRADD_TRAF2	0.00438596	66	Gi	0
28	actNFkB	0.00333333	67	GAP	0
29	actCAPP	0.00333333	68	PI3K	0
30	RIP_RAIDD	0.00254386	69	PIP2	0
31	actCASP8	0.00254386	70	AKT	0
32	actCASP3and9	0.00219298	71	PDK1	0
33	recruitRAIDD	0.00184211	72	CellSurvival	0
34	cIAP2	0.00140351	73	ERK	0
35	actCASP2	0.00131579	74	MEK	0
36	recruitRIP	0.00122807	75	RAF	0
37	BIDinhibition	0	76	RIP	0

38	CASP3n9+cytoCinhibition	0	77	CASP8inhibition	0
39	AKTinhibition	0			

5 Values of Closeness Centrality of the Entities of TNF-α-mediated sphingomyelin signaling pathway model

S.NO.	Name	Closeness Centrality	S.No.	Name	Closeness Centrality
1	actCASP2	1	40	ProteaseCathepsinD	0.33333333
2	actCASP8	1	41	actPI3K	0.33333333
3	cIAP2	1	42	PIP2	0.33333333
4	actCAPP	1	43	Ceramide	0.31578947
5	actCASP3and9	1	44	GiCoupledReceptor	0.30434783
6	actAKT	1	45	CAPK	0.3
7	actERK	1	46	RasGDP	0.3
8	CytochromeC	0.75	47	Sphingosine	0.29126214
9	CASP3and9	0.75	48	TRAF2	0.28571429
10	RIP_RAIDD	0.66666667	49	PI3K	0.27586207
11	CASP2	0.66666667	50	CDase	0.26548673
12	actNFkB	0.66666667	51	GAP	0.25925926
13	CAPP	0.66666667	52	SM	0.25
14	recruitAKT	0.625	53	actSMase	0.24793388
15	PDK1	0.625	54	actS1PR	0.24590164
16	proCASP8	0.6	55	Gi	0.24590164
17	actMEK	0.6	56	SMase_translocated	0.24390244
18	ERK	0.6	57	recruitFADD	0.22
19	recruitRAIDD	0.5	58	S1P	0.20779221
20	recruitRIP	0.5	59	S1PR	0.20779221
21	phIKK	0.5	60	TNFR_TRADD	0.20487805
22	NFkB	0.5	61	TNFR_FAN	0.20394737
23	actBID	0.5	62	SMase	0.20394737
24	Mitochondria	0.5	63	SMS	0.2027027
25	actRAF	0.44444444	64	PKC	0.2012987
26	MEK	0.44444444	65	TNFR	0.19742489
27	actCathepsinD	0.42857143	66	FADD	0.18478261
28	BID	0.42857143	67	SPHK	0.18085106
29	PIP3	0.42857143	68	TNFR_TNF	0.17813765
30	AKT	0.42857143	69	FAN	0.17391304
31	RAIDD	0.4	70	TRADD	0.1733871
32	actNIK	0.4	71	TNFalpha	0.15410959
33	IKK	0.4	72	CellDeath	0
34	RasGTP	0.4	73	CellSurvival	0
35	RIP	0.4	74	CASP8inhibition	0
36	actCAPK	0.35714286	75	BIDinhibition	0
37	RAF	0.35714286	76	CASP3n9+cytoCinhibition	0

38	TRADD_TRAF2	0.33333333	77	AKTinhibition	0
39	NIK	0.33333333			

6 Sensitivity Values of Entities of TNF-α-mediated sphingomyelin signaling pathway model against Interaction Parameters

7 Sensitivity Values of Entities of TNF-α-mediated sphingomyelin signaling pathway model against other Entities

**Interaction_1**	**Interaction_2**	**Interaction_7**	**Interaction_8**	**Interaction_15**	**Interaction_18**	**Interaction_23**	**Interaction_27**	**Interaction_28**	**Interaction_29**	**Interaction_34**	**Interaction_35**	**Interaction_40**	**Interaction_43**	**Interaction_49**	**Interaction_51**
678.9 TNFα	398.9 recruit RAIDD	694.2 FADD	388.4 recruit RAIDD	691.3 FAN	550.8 CAPK	683.6 CAPP	705.1 ProtCathD	220.5 actCASP 3n9	547.7 S1PR	332.8 GAP	422.3 S1PR	436.6 actCAPP	234.7 Cell Survival	696.5 RIP	669.7 Cell Death
647.8 FAN	373.4 RAIDD	597.2 TRAF2	373.9 TRADD	657.6 SMase	472 RAF	669.1 actCAPP	682 BID	220.5 Cell Death	532 Gi	326.8 RasGDP	409.9 Gi	302.1 Cell Survival		599.4 TRAF2	483.3 Cell Survival
613.7 SMase	361.4 TRADD	575.5 IKK	361.4 RAIDD	647.8 TNFα	459.7 MEK	277 ProtCathD	653.4 Mitochondria		258.6 P13K	316.2 RAF	199.1 PI3K			595 recruit RAIDD	
592.3 PKC	280.3 TRAF2	556.1 IKK	290.3 TRAF2	636.3 PKC	449.2 ERK	267.9 BID	641.6 CASP 3n9		250 PIP2	306.6 MEK				577.6 NIK	
592.3 SMase (translocated)	270.2 NIK	537.2 NFkB	280.3 IKK	636.3 SMase (translocated)	295.7 Cell Survival	266.2 RAF	471.4 Cell Death		244.2 AKT	298.4 ERK				577.1 CASP2	
339.7 TRADD	261.2 IKK	453 cIAP2	271.3 IKK	323.9 TRADD	202.2 ProtCathD	258.8 MEK	280.3 RAF		238.5 actCAPP	209.6 Cell Survival				558.2 IKK	
328.3 RAIDD	252.5 NFkB	447.1 SMase	262.5 NFkB	313.1 RAIDD	195.5 BID	254.8 Mitochondria	277 CAPP		237.6 PDK1					539.2 NFkB	
295 recruit RAIDD		431.4 PKC		281.7 recruit RAIDD	192 CAPP	252.5 ERK	272.5 MEK		236.8 RasGDP					418.9 Cell Death	
263.9 TRAF2		431.4 SMase (translocated)		251.1 TRAF2	186.3 Mitochondria	250.2 CASP 3n9	265.9 ERK		217.4 ProtCathD					402.6 cIAP2	
254.8 NIK		239.1 ProCASP8		242 NIK	182.9 CASP 3n9	246.4 Cell Survival	250 actCAPP		210.1 BID						
246.6 IKK				233.9 IKK			239.2 Cell Survival		206.5 CAPP						
238.7 NFkB				226 NFkB			205 S1PR		203.6 Mitochondria						
							202.2 CAPK		201 Cell Survival						
							199.1 Gi		199.8 CASP 3n9						

TNFR	TNFR_TNF	recruit_RIP	RIP_RAIDD	actCASP2	actCASP8	recruit_FADD	TNFR_TRADD	actNFkB	phIKK	actNIK	TRADD_TRAF2	TNFR_FAN	actSMase	actCAPK
28.46 SMase	25.02 TRADD	41.95 recruit_RAIDD	48.78 CASP2	40.07 Cell Death	35.36 Cell Death	34.45 SMase	38.86 TRAF2	39.45 cIAP2	48.54 NFkB	48.51 IKK	48.33 NIK	49.04 SMASE	48.36 PKC	48.29 RAF
28.36 FAN	24.18 RAIDD	40.73 CASP2	38.86 Cell Death			33.3 PKC	37.53 NIK		38.1 cIAP2	47.05 NFkB	46.84 IKK	47.4 PKC	48.36 SMase_translocatd	47.1MEK
27.48 PKC	21.74 recruit_RAIDD	30.99 Cell Death				33.3 SMase (translocated)	36.34 IKK			36.71 cIAP2	45.39 NFkB	47.4 SMase_translocatd		46.09 ERK
27.48 SMase_translocatd	19.43 TRAF2					14.97 proCASP2	35.19 NFkB				35.15 cIAP2			33.77 CellSurvival
20.29 TNFα	18.75 NIK						26.82 cIAP2							
	18.15 IKK													
	17.56 NFkB													

actCASP3n9	CytocromeC	actBID	actCathpsinD	Sphingosine	S1P	actS1PR	GiCoupledReceptor	RasGTP	actPI3K	PIP3	recruitAKT	actAKT	actERK	actMEK	actRAF
40.07 Cell Death	49.21 casp3n9	48.21 Mitochondria	48.47 BID	47.07 S1PR	48.51 S1PR	48.75 Gi	28.15 CellSurvival	43.93 RAF	48.61 PIP2	49.05 AKT	48.81 PDK1	32.16 CellSurvival	37.22 CellSurvival	48.98 ERK	48.8 MEK
	39.28 Cell Death	47.43 casp3n9	46.64 Mitochondria	45.82 Gi	47.26 Gi	27.21 CellSurvival	24.36 PI3K	42.85 MEK	47.67 AKT	47.89 PDK1	29.91 CellSurvival			36.3 CellSurvival	47.78 ERK
		37.47 Cell Death	45.86 casp3n9	25.4 CellSurvival	26.22 CellSurvival	23.74 PI3K	23.67 PIP2	41.93 ERK	46.55 PDK1	28.32 CellSurvival					35.22 CellSurvival
			35.88 Cell Death	22.3 PI3K	23 PI3K	23.05 PIP2	23.21 AKT	30.64 CellSurvival	26.69 CellSurvival						
				21.62PIP2	22.32 PI2	22.59 AKT	22.66 PDK1								
				21.16 AKT	21.86 AKT	22.06 PDK1	22.52 RasGDP								
				20.65 PDK1	21.34 PDK1	21.9 RasGDP	21.91 RAF								
				20.45 RasGDP	21.15 RasGDP	21.29 RAF	21.37 MEK								
				19.92 RAF	20.55 RAF	20.75 MEK	20.9								
				19.39 MEK	20.01 MEK	20.29 ERK									
				18.93 ERK	19.55 ERK										
