# Supplementary material for: System level modeling and analysis of TNF-α mediated sphingolipid signaling pathway in neurological disorders for the prediction of therapeutic targets
Source: Front Physiol. 2022 Aug 19;13:872421. doi: 10.3389/fphys.2022.872421 (PMC9437628; doi:10.3389/fphys.2022.872421)
Supplement: Supplementary file 2 [file DataSheet1.PDF]

1 SUPPLEMENTARY DATA

1.1 Figures

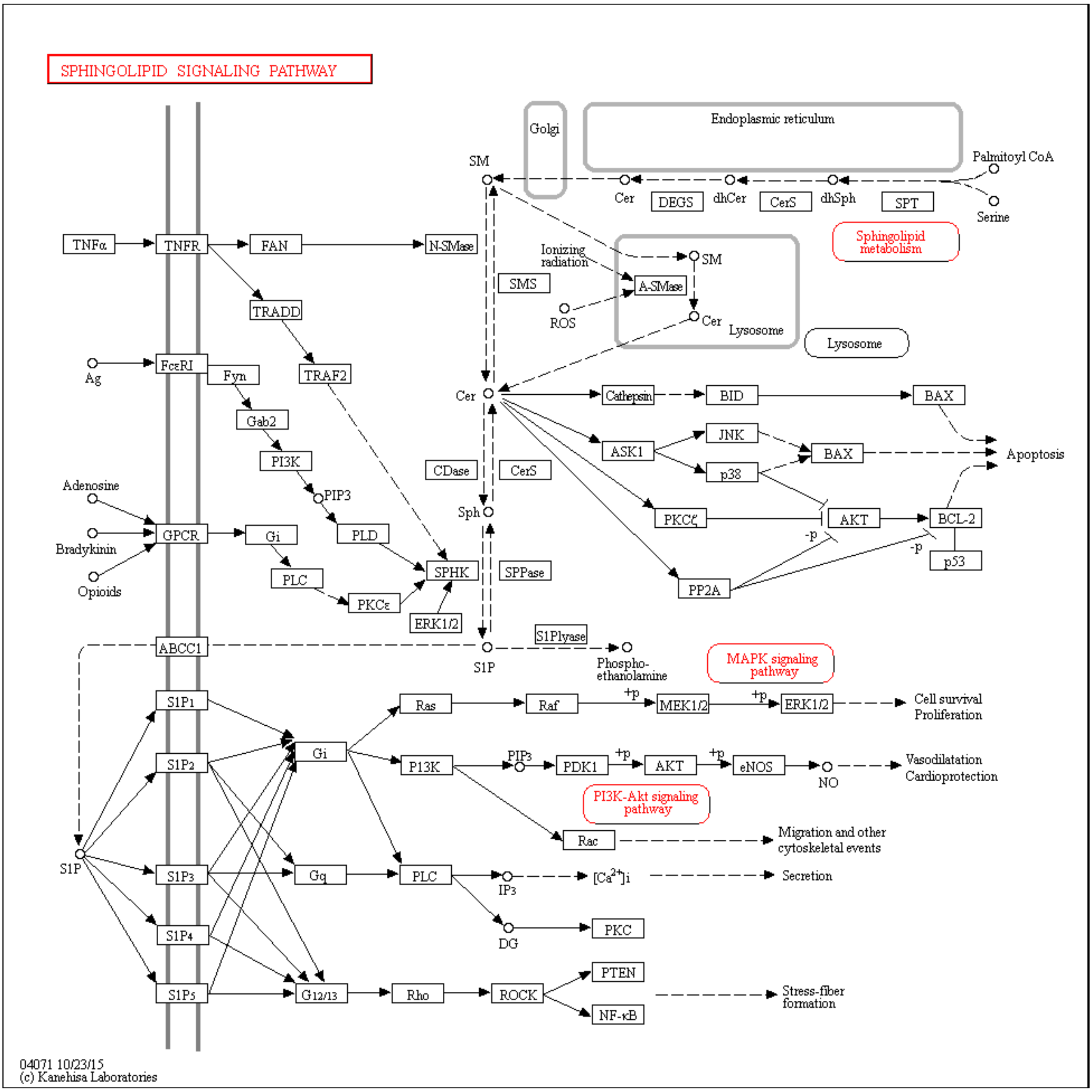

Figure S1. KEGG model of Sphingolipid Signaling Pathway- The sphingolipid metabolism is associated with the variety of cellular signaling pathways. It is regulated by an upstream TNF-α signaling cascade at various levels. Ceramide and S1P are the potent bioactive sphingolipid metabolites that regulate cellular responses to stress by generating opposing effects. S1P promotes cell growth and survival mechanisms andalso acts as a ligand for G-protein coupled receptors. Ceramide triggers intrinsic and extrinsic apoptotic pathways via receptor-independent mechanisms.

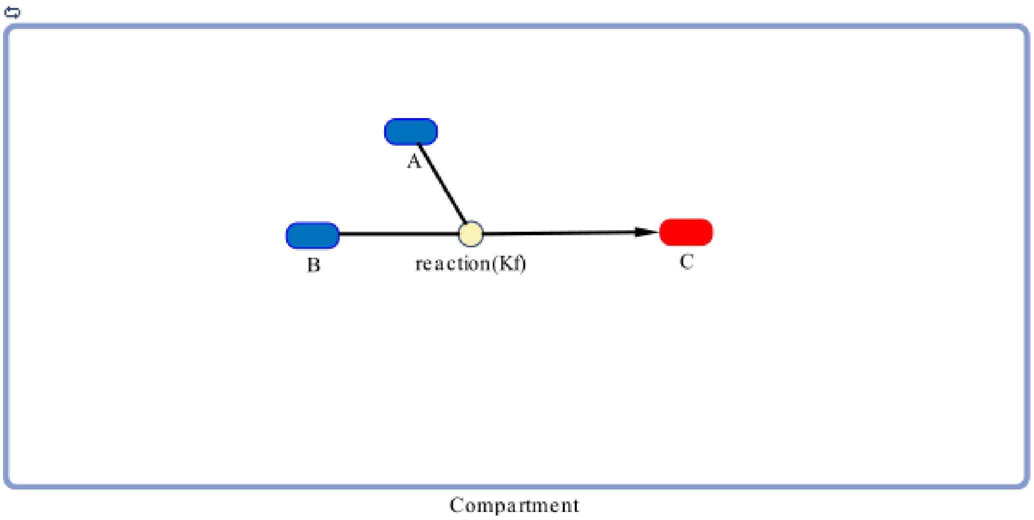

(a)

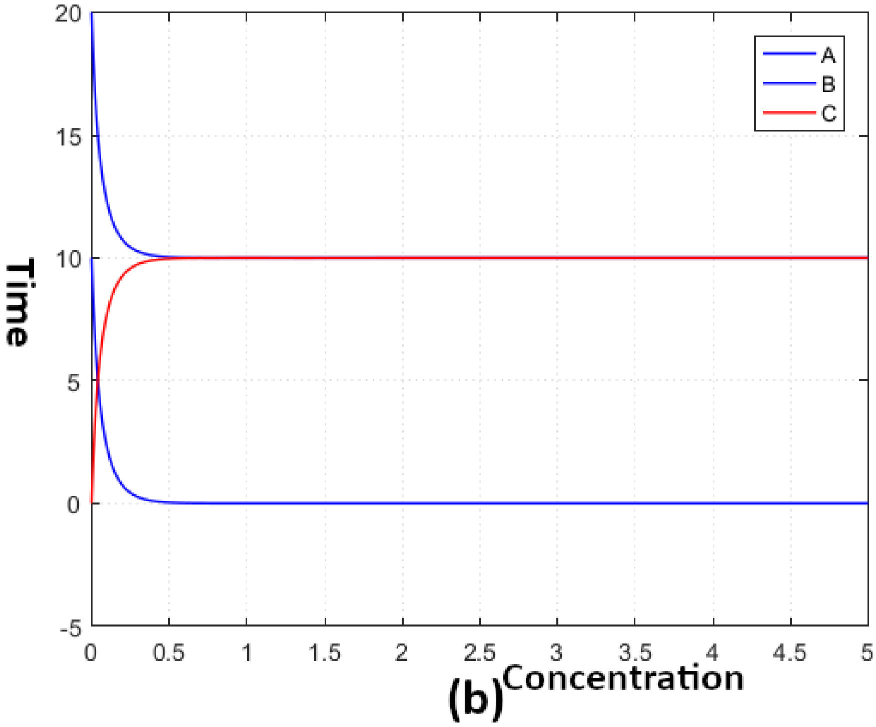

(b)

Figure S2. (a) Simbiology Model Representation for Example interaction (represented by Reaction 1)- Species and interactions are represented by rectangles and circles respectively. Edges are used to connect species with interactions. (b) **Simulation of Example interaction (Reaction 1)-** Blue curves show the simulation of reactants A and B whereas red curve represents the simulation of product C.

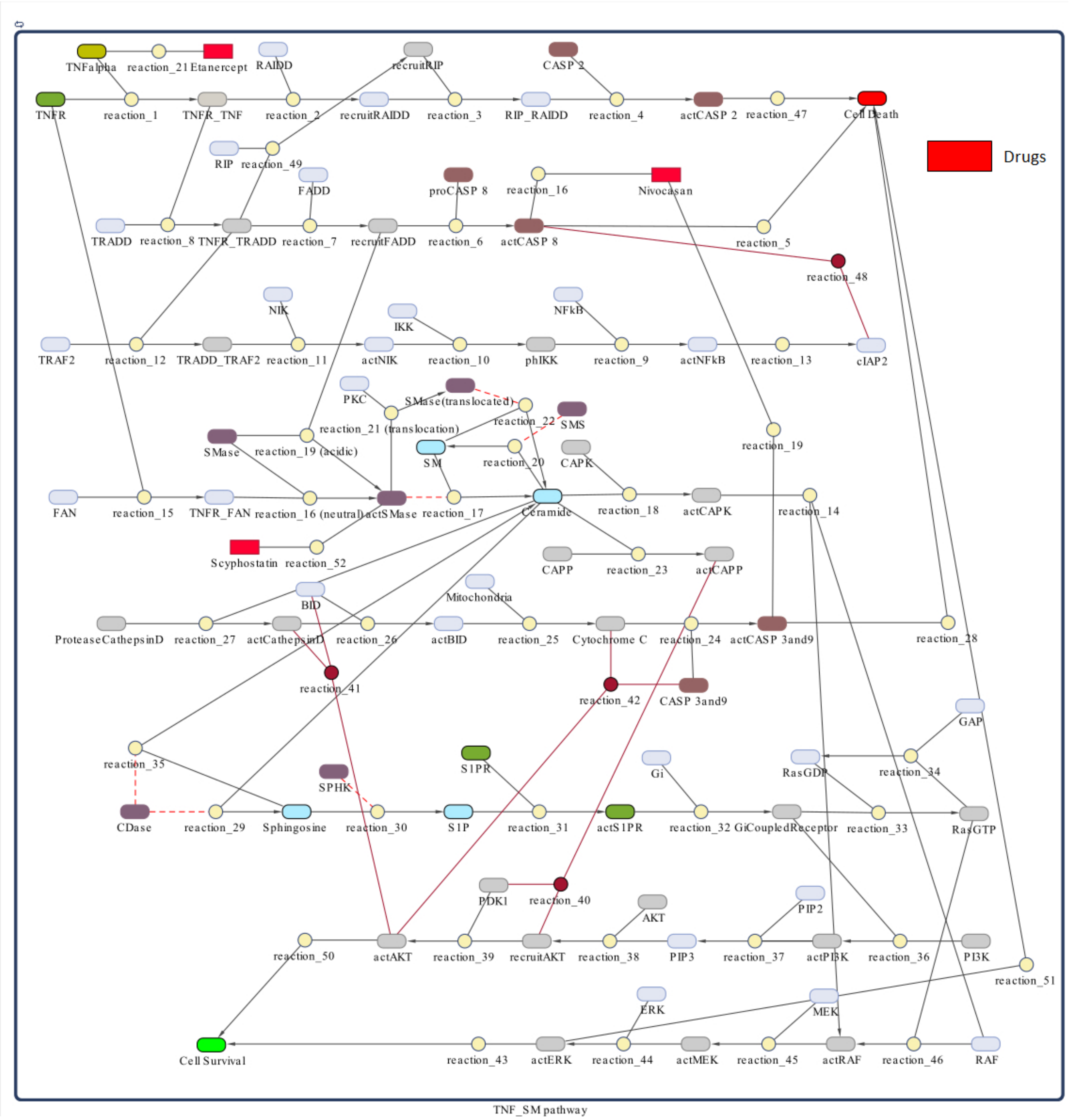

Figure S3. TNF-α mediated sphingomyelin signaling pathway model with drugs and their corresponding site of action- The drugs are represented by squares having dark pink color. All drugs follow inhibitory mode of action.

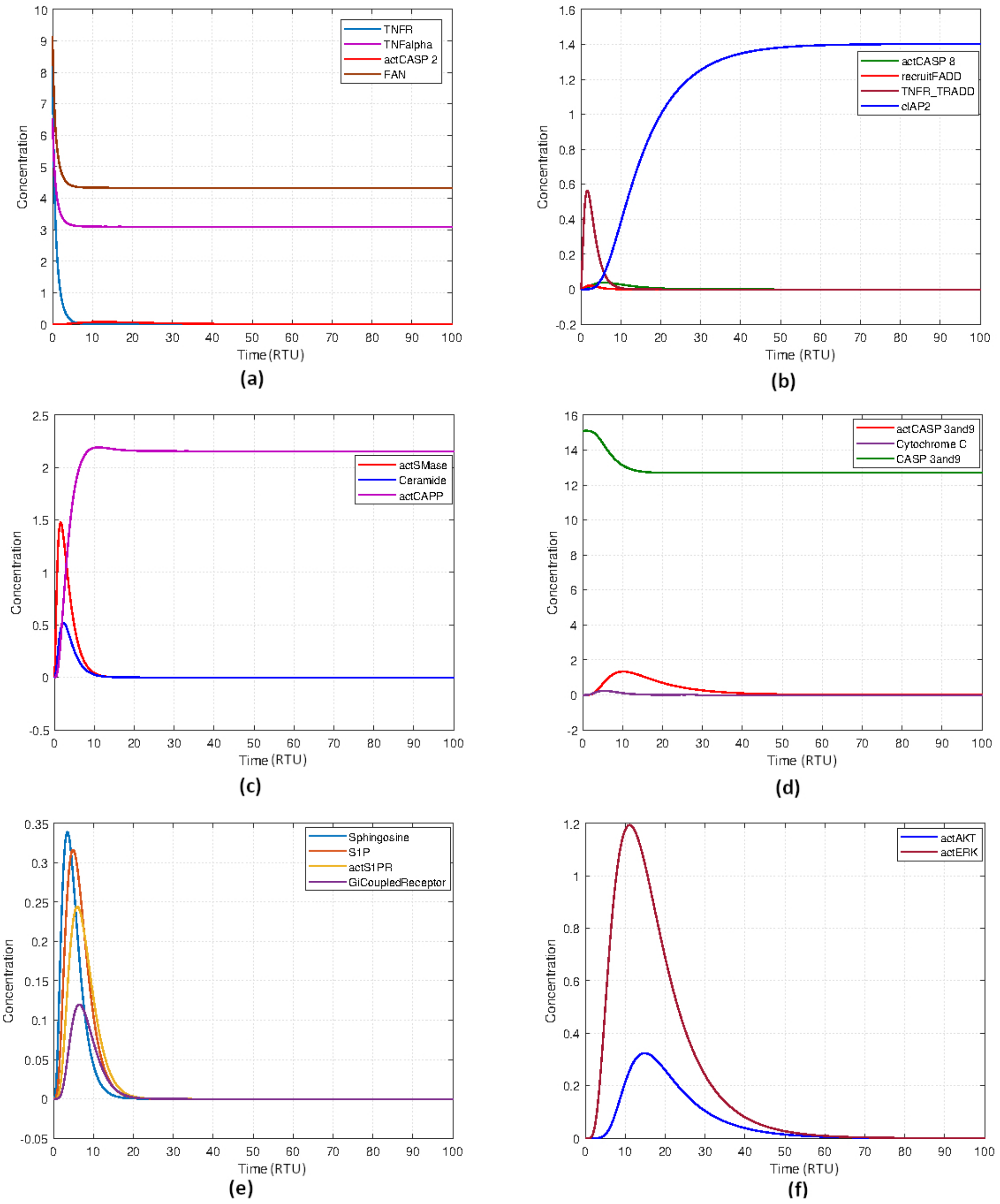

Figure S4. Simulation of entities at the balanced state of neuronal survival and apoptosis-(a) Simulation showing TNF-α binding with TNFR. (b) Simulation showing formation of TNFR_TRADD , FADD recruitment, Caspase 8 activation, and activation/ inhibition of cIAP2. (c) Simulation of Ceramide, SMase and CAPP. (d) Simulation showing Cytochrome C release and activation of Caspases 3 and 9. (e) Simulation showing formation of sphingosine and S1P along with the stimulation of Gi Coupled receptor. (f) Simulation showing AKT activation and ERK activation.

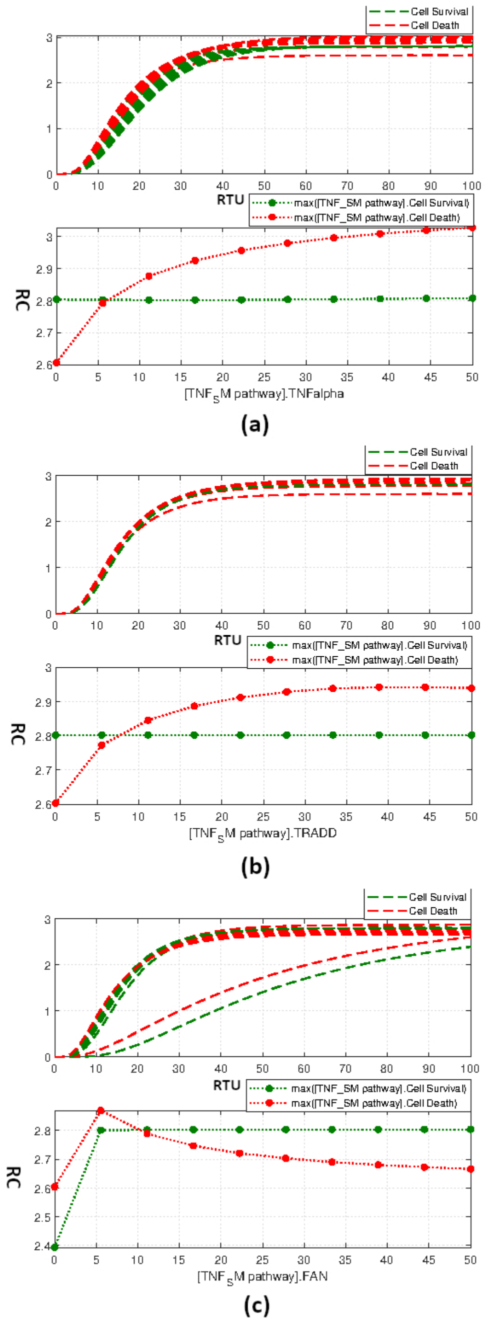

Figure S5. Scanning of Input Entities TNF-α, TRADD, and FAN- (a) Effect of TNF-α Concentration on Neuronal Survival and Neuronal Death. Increasing TNF-α concentration over the range 0-50 enhances the neuronal apoptosis without impacting neuronal survival **(b) Effect of TRADD Concentration on Neuronal Survival and Neuronal Death.** Increasing TRADD concentration over the range 0-50 enhances the neuronal apoptosis without impacting neuronal survival **(c) Effect of FAN Concentration on Neuronal Survival and Neuronal Death.** Increasing FAN concentration over the range 0-50 enhances the neuronal apoptosis up to the concentration of 5 and afterward shows a gradual decrease in trend with an increase in quantity. However, increasing FAN concentration over the range 0-50 enhances the neuronal survival up to the concentration of 5 and afterward shows no pronounced effect with an increasing quantity.

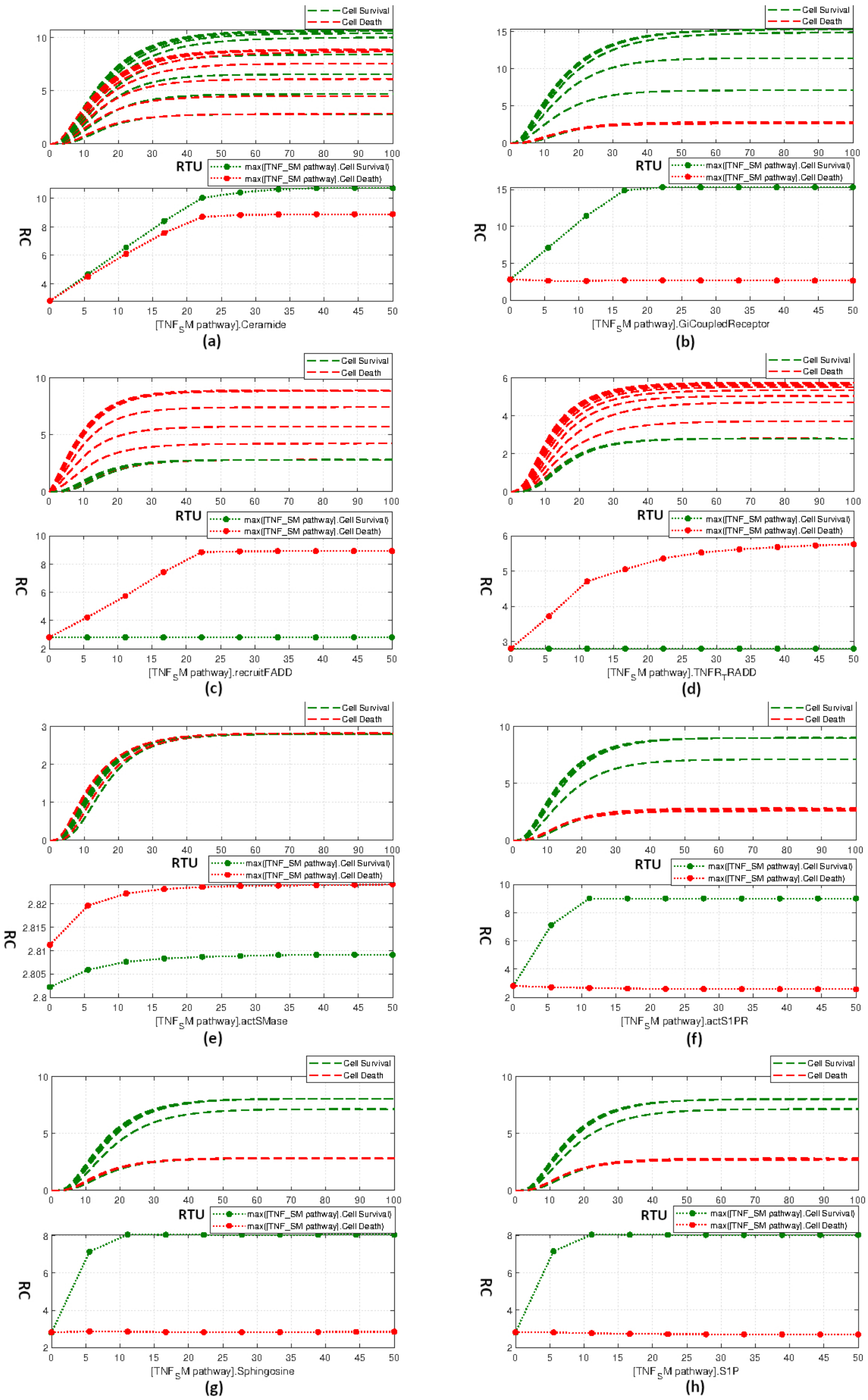

Figure S6. Scanning of Entities with the High betweenness centrality- (a) Scanning of Ceramide. (b) Scanning of Gi-Coupled Receptor. (c) Scanning of recruited FADD. (d) Scanning of TNFR_TRADD complex. (e) Scanning of actSMase. (f) Scanning of actS1PR. (g) Scanning of Sphingosine. The ceramide and actSMase impact both neuronal survival and neuronal apoptosis with an increasing concentration up to the value of 23 and 12, respectively. Gi-Coupled Receptor, actS1PR, Sphingosine, and S1P cause enhanced neuronal survival with increasing quantities without impacting neuronal apoptosis. However, increasing recruited FADD and TNFR_TRADD concentrations over the range 0-50 show an increasing influence on neuronal apoptosis.

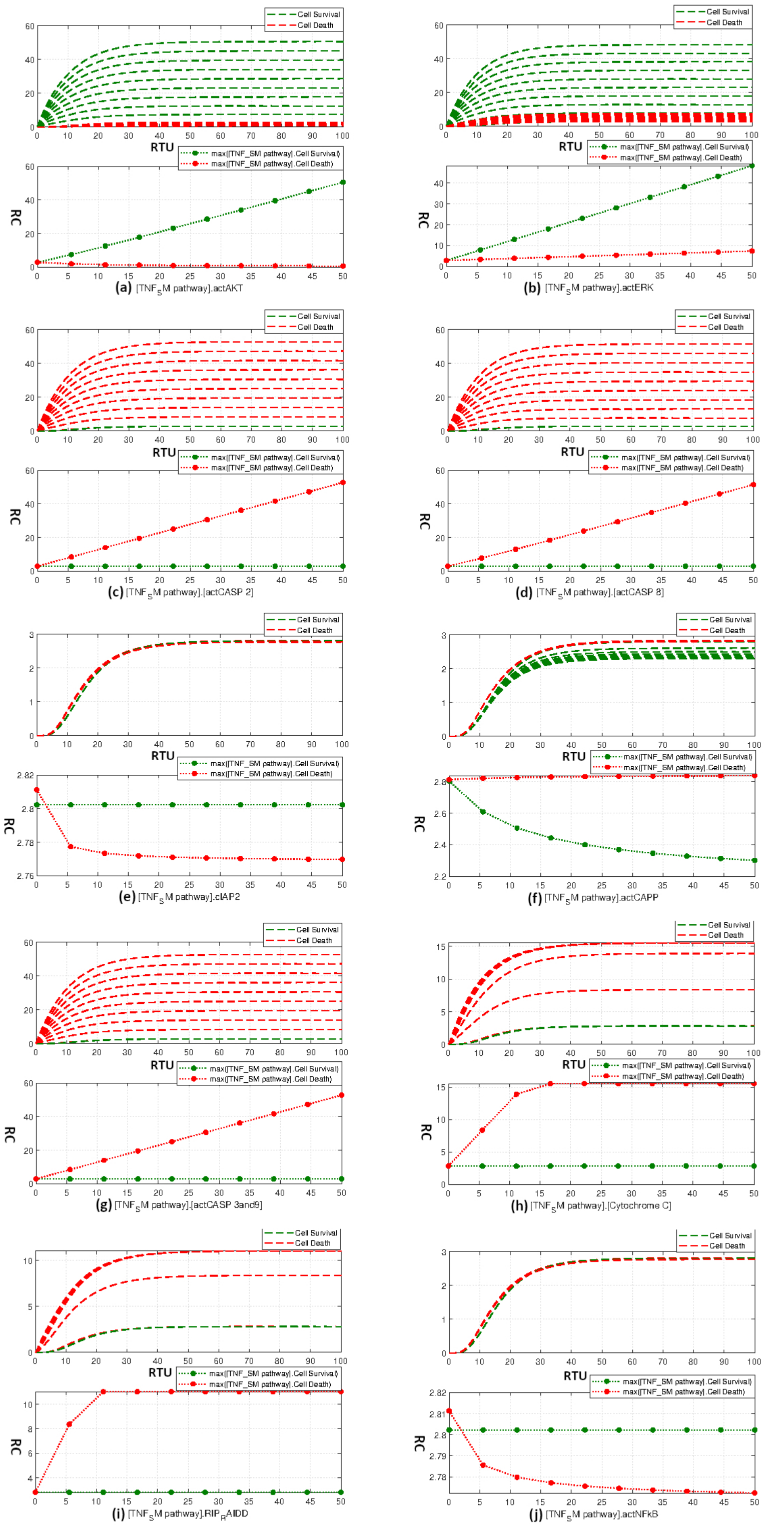

Figure S7. Scanning of Entities with the High closeness centrality (a) Scanning of activated AKT. (b) Scanning of activated ERK. (c) Scanning of actCASP2. (d) Scanning of actCASP8. (e) Scanning of cIAP2. (f) Scanning of actCAPP. (g) Scanning of actCASP 3and9. (h) Scanning of Cytochrome C. (i) Scanning of RIP_RAIDD. (j) Scanning of actNFκB. The increasing actAKT and actERK over the range 0-50 have an increasing influence on neuronal survival while increasing actCAPP concentration over the range 0-50 negatively impacts neuronal survival. The increase in the concentration of the entities actCASP2, actCASP8, actCASP 3and9, Cytochrome C, and RIP_RAIDD over the range 0-50 enhances neuronal apoptosis. However, an increase in the concentration of the entities cIAP2 and actNFκB over the range 0-50 reduces neuronal apoptosis without impacting neuronal survival.
